# A Rigid–Soft Graded Organic–Inorganic Interlayer for Durable and Corrosion-Resistant Zinc Anodes

**DOI:** 10.1007/s40820-025-02020-8

**Published:** 2026-01-05

**Authors:** Zhiyu Wang, Junlun Cao, Zixuan Yang, Jianli Cheng, Dan Liu, Weiwei Lei

**Affiliations:** 1 School of Science, RMIT University, Melbourne, VIC 3000 Australia; 2 Institute for Frontier Materials, Deakin University, Waurn Ponds Campus, Locked Bag 20000, Melbourne, VIC 3220 Australia; 3 School of Optoelectronic Science and Engineering, University of Electronic Science and Technology of China, Chengdu, 611731 People’s Republic of China

**Keywords:** Liquid plasma oxidation, Zinc oxide, Hybrid interfacial layer, Zinc anode, Aqueous zinc-iodine battery

## Abstract

Aqueous zinc (Zn)-ion batteries hold great promise as renewable energy storage system for carbon–neutral energy transition. However, Zn anodes suffer from poor Zn plating/stripping reversibility due to Zn dendrite growth and side reactions. Existing Zn interfacial modification strategies based on single-component or homogeneous structure are insufficient to address these issues comprehensively. Herein, we rationally designed an organic–inorganic hybrid interfacial layer with rigid-to-soft graded structure for dendrite-free and stable Zn anodes. A liquid plasma-assisted oxidation technology is developed to rapidly construct a porous ZnO inner framework in situ. This ZnO layer offers high interfacial energy, mechanical robustness, and an open structure that facilitates ion transport while firmly anchoring a subsequently coated soft polymer layer. The resulting architecture presents a structurally graded and functionally complementary interface, enabling effective dendrite suppression, continuous Zn ion transport, and enhanced corrosion resistance. As a result, a long cycling stability of more than 6000 h can be achieved at 1 mA cm^−2^ for 1 mAh cm^–2^ in symmetric cells. When used as anodes for zinc-iodine full battery, the hybrid interlayer can effectively prevent the Zn anodes from the corrosion by polyiodine, enabling stable cycling and negligible capacity decay (~ 0.02‰ per cycle) for over 10,000 cycles at 2.0 A g^−1^. This work demonstrates a promising interfacial design strategy and introduces a novel liquid plasma-assisted oxidation route for fabricating high-performance Zn anodes towards next-generation aqueous batteries.

**Supplementary Information:**

The online version contains supplementary material available at 10.1007/s40820-025-02020-8.

## Highlights

- A hybrid interfacial layer with a rigid-to-soft graded structure and functionally complementary composition.
- A facile and scalable liquid plasma-assisted oxidation process for preparing the porous ZnO inner layer.
- Good cycling stability of zinc anodes for more than 6,000 h at a current density of 1 mA cm^−2^ for 1 mAh cm^−2^ and ultra-low capacity decay (~0.02‰ per cycle) for over 10,000 cycles for zinc-iodine battery.

**Supplementary Information:**

The online version contains supplementary material available at 10.1007/s40820-025-02020-8.

## Graphical abstract

**Figure:**
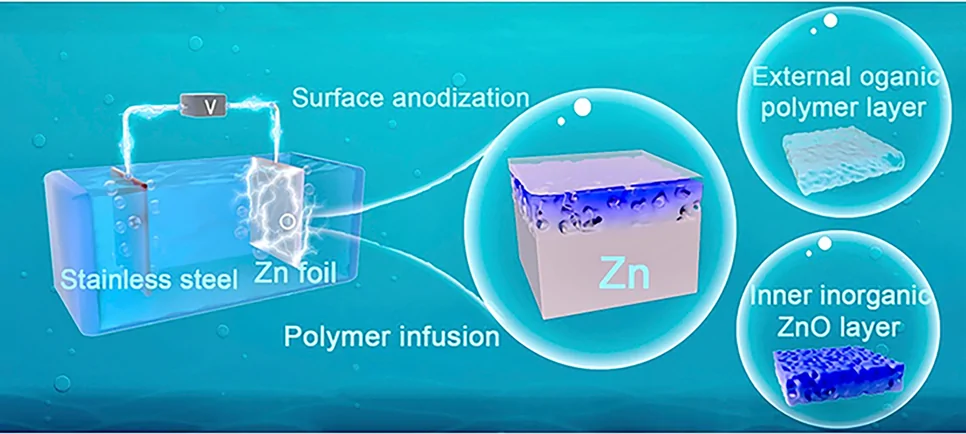

## Introduction

Metallic zinc (Zn) has been regarded as a promising anode material for aqueous batteries due to its high theoretical capacity (820 mAh g^−1^), low electrochemical potential (− 0.762 V vs. standard hydrogen electrode (SHE)), good environmental sustainability, and low cost [1–3]. However, Zn anodes suffer from intrinsic unstable surface caused by dendrite formation, corrosion, and side reactions such as hydrogen evolution reaction (HER), leading to poor cycling irreversibility and low Coulombic efficiency (CE). To this end, several strategies have been proposed to address these issues, including Zn anode interphase engineering, electrolyte configuration and additives optimization, and manipulation of Zn nucleation and growth [4–8]. The primary goal of these strategies is to regulate the interphase between Zn metal and electrolyte to ensure stable Zn nucleation and deposition, fast ion transport, and effective side reaction suppression. Therefore, the interfacial engineering of Zn anodes has garnered significant attention for its capability to effectively, controllably, and scalably modify the Zn anode interface [9, 10]. In addition, this strategy demonstrates excellent compatibility, enabling the resulting surface-protected Zn anodes to be widely utilized in various types of Zn-based batteries when paired with appropriate cathodes and electrolytes [11].

Recent literature suggests that the state-of-the-art interfacial layers for Zn anodes should possess: (1) high mechanical robustness to suppress dendrite formation, (2) high interfacial energy to adapt to interfacial repositioning during anode volume change, (3) high ion conductive and low electronic conductivity to support fast and even ion transport, and (4) strong corrosion resistance to suppress side reactions [12–17]. To achieve these design principles, a variety of interfacial layers based on both inorganic and organic materials with different structures have been extensively investigated. Generally, organic material-based interfacial layers, such as poly(vinyl butyral) [18] and polyimide [19], feature easy processing and good scalability and can effectively improve the ion transport and surface stability of Zn anode. However, the soft nature, poor mechanical properties, and strong adhesion of these organic interfacial layers to Zn anode surface lead to their breakage during Zn dendrite formation. Inorganic materials with high modulus, such as ZnF_2_ [20], Zn_3_(PO_4_)_2_ [21], ZnSe [22], and ZnO [23], have been formed on the surface of Zn anodes via methods like hydrothermal reaction and chemical vapour deposition to stabilize Zn anode surface. These inorganic materials can provide fast ion transport channels and physical confinement for Zn growth. However, their inherent rigidity and loose structure result in cracking and detachment from the Zn surface during cycling, diminishing their effectiveness in suppressing dendrite formation and side reactions.

Faced with the dilemma, developing new hybrid interfacial layers with carefully designed structure and optimized organic/inorganic composition offers a promising solution to meet these multiple design principles essential for stable Zn anodes [24–26]. Recent studies on metal-based anodes indicate that an inorganic inner layer with high modules and interfacial energy can effectively suppress the dendrite formation and self-reposition at the interface during volume change, while an organic external layer can provide continuous ion transport, prevent side reactions, and further improve the structural integrity and mechanical performance of the entire hybrid interlayer [14]. However, designing such a hybrid interfacial layer with complementary functions requires precise control over its structure and composition, with the inorganic layer playing essential role in determining the structural and compositional design of the whole hybrid layer.

ZnO has emerged as a promising candidate for the inorganic inner layer due to its good chemical stability, high interfacial energy (197.16 meV Å^−2^), high modules (130 GPa), zincophilic property, electron-insulating nature, hydrophobic property, and low cost [15, 27, 28]. Despite various efforts to construct ZnO interlayers with different morphologies and structures to protect the Zn anode, not surprisingly, Zn anodes modified with solely ZnO-based layer still exhibit unsatisfactory electrochemical performance due to insufficient interfacial stability [27, 29, 30]. In addition, the commonly employed fabrication techniques, such as hydrothermal synthesis, are typically energy- and time-intensive, involve complex fabrication processes, and often raise environmental concerns [23]. Moreover, these methods also suffer from poor control over film morphology, uniformity, and limited adhesion strength, leading to compromised overall anode performance. Therefore, a simple, effective, environmentally friendly, and scalable technique for the construction of ZnO-based interlayer with high uniformity and adhesion is highly required for the development of ZnO-based hybrid interfacial layers. Liquid plasma-assisted anodization technologies have been widely used to prepare metal oxides for various applications including photocatalysis and corrosion resistance [31], which can generate uniform coatings with unique microstructures that are difficult to achieve by conventional methods. Therefore, liquid plasma-assisted anodization offers a highly promising and yet underexplored strategy for the fabrication of porous ZnO interfacial layers, potentially enabling significant improvements in zinc ion battery performance.

Herein, we report the rational design of a structurally graded and functionally complementary organic–inorganic hybrid interfacial layer to stabilize Zn anodes. This hybrid interface features a rigid-to-soft architecture, compromising in situ*-*formed porous ZnO as an inner layer and ex situ*-*coated polyvinyl alcohol (PVA) as an external layer. The ZnO inner layers, generated through a facile, ultrafast, and environmentally friendly liquid plasma-assisted anodization process, can effectively modulate Zn ion transport, regulate Zn nucleation behaviour, and suppress Zn dendrite formation, owing to its high interfacial energy, electron-insulating nature, and strong interaction with Zn ion. In addition, its open porous structure provides a mechanically robust scaffold that firmly anchors the ion-conducting PVA external layer onto Zn anode within its robust skeleton, forming a structurally integrated hybrid interlayer with strong adhesion. The soft PVA external layer can further homogenize ion transport and suppress side reactions. Experimental and computational results show that the as-designed ZnO and PVA hybrid interfacial layer can synergistically regulate Zn nucleation, homogenize the electrical filed, facilitate Zn ion desolvation, and suppress Zn dendrite formation, leading to much more improved cycling stability and corrosion resistance than ZnO-modified Zn and pristine Zn anodes. Taking advantage of the hybrid interfacial layer, an exceptional cycling stability of more than 6000 h can be achieved at 1 mA cm^−2^ for 1 mAh cm^−2^. Moreover, when paired with iodine (I_2_) cathodes, the hybrid interfacial layer can effectively protect the Zn anode from the corrosion of polyiodine, enabling a long cycling stability of more than 10,000 cycles for Zn-I_2_ full cells.

## Experimental Section

### Electrode Preparation

KOH solutions with a concentration of 0.1 M were prepared as electrolyte for anodic oxidation. Commercial Zn foil (0.1 mm × 60 mm × 40 mm) with one side taped by Kapton tape was connected to the anode and a stainless-steel mesh connected to the cathode of a TruPlasma DC 4010 G2 DC Power Supply. 10 wt% PVA dispersion (Mw. 146–186 K) was blade-coated on the surface-treated Zn and neat Zn foils. An air plasma treatment of PVA-coated Zn foils was conducted with argon as reactive gas under an output powder of 200 W in atmosphere for 30 s. All the electrodes were punched into discs with a diameter of 11 mm.

The cathode was prepared by mixing I_2_ and carbon black (CB) with a mass ratio of 6:4, followed by thoroughly grinding for 1 h. The mixed I_2_ and CB (I_2_@CB) were heat-treated at 80 °C for 8 h. The I_2_ cathodes was prepared by mixing 90 wt% of I_2_@CB and 10 wt% PVDF in 1-methyl-2-pyrrolidone (NMP) to form a homogeneous slurry, followed by coating on titanium foil and vacuum-drying for 12 h at 50 °C. A polyurethane (PU)-based interfacial layer was further prepared by blade-coating a 10 wt% PU in dimethylformamide (DMF) on the surface of the as-prepared I_2_@CB cathode and dried for 12 h at 50 °C in vacuum. The active material loading of the cathode was 1.5–2 mg cm^−2^ and the PU layer was ~ 0.1 mg cm^−2^. For pouch cells, all the electrodes were cut into 3 cm × 3 cm pieces, and the active material loading of the cathode was 5–5.5 mg cm^−2^. The polyiodine solution was prepared by dissolving I_2_ and KI with a molar ratio of 1:100 in deionized water.

### Characterization

Scanning electron microscopy (SEM) images were taken on a Zeiss Supra VP55 at an accelerating voltage of 5 kV. X-ray diffraction (XRD) patterns were obtained on a PANalytical X’pert Powder diffractometer with a CuKα radiation source operated at 40 kV (*λ* = 1.5406 Å). X-ray photoelectron spectroscopy (XPS) data were obtained using a Kratos AXIS Supra instrument. Scratch resistance was evaluated using an Anton Paar NHT + MCT nanoindenter system. Surface roughness was measured by Tencor P16 surface profiler.

### Electrochemical Tests

Symmetric coin cells were assembled in 2032 coin cells with identical Zn or modified Zn as working electrodes and counter (reference electrode), Whatman GF/D glass fibre membrane as separator, and 100 μL of 2 M Zn(CF_3_SO_3_)_2_ as electrolyte, unless otherwise specified. CE and cycling stability test were performed using Cu foil as working electrodes on Land Battery tester. Zn nucleation overpotential was measured by plating Zn at a current density of 0.1 mA cm^−2^. Electrochemical impedance spectroscopy (EIS) was implemented within the range of 10^5^–10^−2^ Hz. Chronoamperogram (CA) curve was obtained by applying a constant potential of − 150 mV (Zn^2+^/Zn) in symmetric cells. Linear sweep voltammetry (LSV) was conducted in a three-electrode configuration in 1 M Na_2_SO_4_ aqueous electrolyte at a scan rate of 5 mV s^−1^ from − 1.1 to − 2.0 V (Ag/AgCl) with bare Zn, ZnO-Zn and PZnO-Zn as working electrodes, graphite rod as counter electrode, and Ag/AgCl as reference electrode. Linear polarization was conducted at a scan rate of 5 mV s^−1^ in symmetric cells from − 0.1 to 0.1 V (Zn^2+^/Zn). Zn nucleation overpotential, CA, LSV, and EIS measurements were taken on a Bio-Logic VMP-300 electrochemical workstation. Zn-I_2_ full cells were assemble with I_2_@CB as cathode, Zn or modified Zn as anode, and 100 μL of 2 M Zn(CF_3_SO_3_)_2_ as electrolyte. Zn-MnO_2_ full cells were assembled with MnO_2_ (4 mg cm^−2^) as cathode, Zn or modified Zn as anode, and 100 μL of 2 M ZnSO_4_ as electrolyte.

The surface capacitance was measured using symmetric cells at a scan rate of 10–20 mV s^−1^ with a voltage range from − 15 to 15 mV (Zn^2+^/Zn). The capacitance is calculated according to the formula (1):

1 $$C \, = \, i/v$$

where *i* is the half difference between positive and negative scanning currents at 0 V (Zn^2+^/Zn) and *v* is the scan rate.

The exchange current densities are calculated according to Eq. (2):

2 $$i \approx i_{0} \frac{F}{ RT}\frac{{\eta_{{{\mathrm{tot}}}} }}{2}$$

where *i*_*o*_ is exchange current, *i* is the measured current, *F* is Faraday constant, *R* is the universal gas constant, *T* is the absolute temperature, and *η*_tot_ is the voltage difference between the plating and stripping.

### Electrical Field Simulation

COMSOL Multiphysics 6.2 software was employed under the “Tertiary current distribution” interface and the “transient with initialization”. The electric field and Zn^2+^ concentration distributions at the anode and electrolyte interfaces were simulated in a simplified two-dimensional model (7 μm × 8 μm) through the finite element method. The physical models used in the simulations are based on scanning electron microscopy results. The electric field migration and equilibrium potential at the electrode surface follow the equations of the Nernst–Einstein relationship. The electrode reaction kinetics follow the Butler–Volmer approximation equation. The electrode surface was represented by constructing irregular blocks to mimic the ZnO with open structure. The initial concentration of Zn^2+^ in the electrolyte was 500 M, and the current densities were set at 225 mA cm^−2^ in both cases. The diffusion coefficient of Zn^2+^ in the electrolyte was set at 3.3 × 10^−10^ m^2^ s^−1^. The operating temperature was 298 K, and the output time step time was set to (0, 0.1, 1) with unit “s”.

### Density Functional Theory Calculations

All density functional theory (DFT) calculations were carried out in the Gaussian16 software package. Geometric optimization and frequency calculations were carried out at the B3LYP-D3(BJ)/def2-TZVP level. The binding energies (E_B_) are calculated according to Eq. (3):

3 $$E_{B} = \, E_{{({\mathrm{complex}})}} {-} \, E_{{{\mathrm{(Zn}}^{{2 + }} {)}}} {-} \, E_{{({\mathrm{frag}})}}$$

where *E*_(complex)_ is the total energy of the complex (Zn^2+^-PAV), *E*_(frag)_ is the energy of each fragment.

## Results and Discussion

### Design and Characterizations of Graded-Structured ZnO-PVA Hybrid Interfacial Layer

**Fig. 1:**
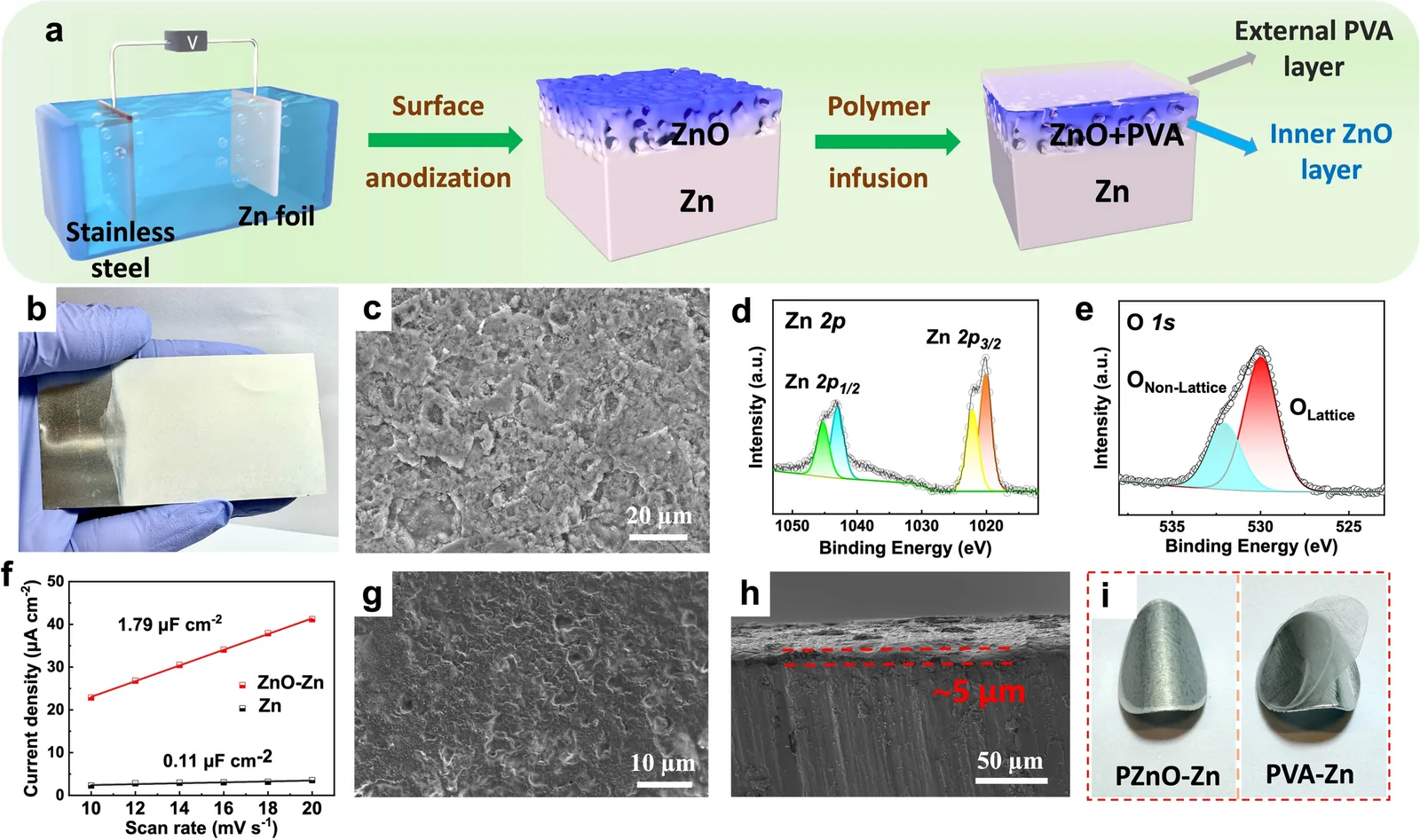
Design strategy and characterizations of PZnO-Zn. **a** Schematic of the preparation of Zn anode with hybrid interfacial layer. **b** Digital image of ZnO-Zn. **c** Top-view SEM image of ZnO-Zn. **d**, **e** High-resolution of **d** Zn 2*p* and **e** O 1*s* spectra of ZnO-Zn. **f** Calculated capacitance of ZnO-Zn and Zn. **g** Top-view and **h** cross-sectional SEM images of PZnO-Zn. **i** Digital images of PZnO-Zn and PVA-Zn electrodes after bending

The design and preparation of the organic–inorganic hybrid interfacial layer with a graded rigid-to-soft structure are achieved through a sequential fabrication strategy, naturally creating a gradual transition in composition and mechanical modulus, as schematically illustrated in Fig. 1a. For the inner layer of the hybrid interfacial layer, which maintains direct contact to the anode surface and need to adapt to electrode surface changes, ZnO was selected as the building block due to its electron-insulating nature, high interfacial energy, and high bulk modulus. Unlike currently reported ZnO-based interlayers prepared by multistep reactions [23, 32], a fast and scalable liquid plasma oxidation technology was proposed and designed to prepare ZnO-modified Zn (ZnO-Zn). A KOH solution with a concentration of 0.1 M was selected as the electrolyte, since dilute electrolytes require higher voltage and lead to rough surface, whereas more concentrated electrolytes generate large currents that suppress voltage rise and bubble formation (Fig. S1). NaOH at a similar concentration was also found to enable effective anodization (Fig. S2). In this work, unless otherwise stated, 0.1 M KOH was used as the electrolyte.

Given that the liquid-phase oxidation process is a dynamic competitive reaction between surface etching and oxidation [33], a wide range of oxidation voltage was initially investigated. At a low voltage between 10 and 20 V, holes and valleys with uneven size distribution can be observed on the surface of Zn foil (Fig. S3a). X-ray diffraction (XRD) results indicated that no new peaks were observed (Fig. S3b), suggesting that the dominant reaction occurring within this voltage range is surface etching of Zn foil. When the voltage was increased to 40–60 V, a uniformly distributed white layer can be formed on the surface of Zn foils in less than 30 s (Fig. 1b), accompanied by vigorous bubbling and significant heat generation. This indicates that the surface treatment process is both facile and scalable. The top-view scanning electron microscopy (SEM) image revealed that the treated Zn foils exhibited a uniform surface with open structure, characterized by the presence of pores and valley-like features (Fig. 1c). Energy-dispersive X-ray (EDX) mapping identified Zn and O elements as the dominant elements in the white-coloured layer, in contrast to the weak O signal observed on pristine Zn foil (Fig. S4). New peaks at 31.7° and 34.4° of the XRD pattern indicated that the main component of the oxidation layer is ZnO (JCPDS No. 36-1451) [34], as shown in Fig. S5. X-ray photoelectron spectroscopy (XPS) revealed that the ZnO-Zn anodized between 40 and 60 V exhibited a secondary Zn 2*p* peak compared with pristine Zn foil (Figs. 1d and S6a), which can be attributed to the presence of Zn–O polar bonds at the interphase of ZnO and Zn metal [35, 36]. Twisting experiments confirmed the strong adhesion between the ZnO layer and the Zn foil (Fig. S7). Consistently, the O 1*s* spectrum showed the peak of lattice-oxygen at approximately 530.0 eV (Fig. 1e), confirming the formation of ZnO. As a control, the O 1*s* spectrum of bare Zn foil does not display this peak (Fig. S6b). Therefore, the dominant chemical reaction at the increased voltage can be attributed to the affinity of Zn for oxygen-containing anions [37], as described in Eq. (4):

4 $${\text{Zn }} + {\text{ 2OH}}^{ - } - {\text{ 2e }} = {\text{ ZnO }} + {\text{ H}}_{{2}} {\mathrm{O}}$$

The formation of ZnO is promoted by heat and gas generated during the vigorous plasma anodic reaction, which disrupts the close contact between Zn and the electrolyte and simultaneously accelerates the oxidation process [38]. Moreover, the voltage instability and fluctuations observed in this voltage range indicate the occurrence of micro-discharges or localised breakdowns at the gas–liquid interface, which could be caused by the liquid-phase plasma [39]. This is further supported by the intense generation of heat and gas bubbles observed at elevated voltages (90 and 150 V), where micro-arc was even observed at 150 V or above, confirming the occurrence of liquid-phase plasma at the interface. Correspondingly, the ZnO layer oxidized at these elevated voltages underwent notable morphological evolution, with ZnO becoming increasingly porous (Fig. S8). However, partial delamination of ZnO can be observed on the surface of Zn foils treated at these voltages (Fig. S9). In contrast, at a low voltage, most Zn atoms dissolve from the Zn foil into electrolyte and react with surrounding –OH^−^ ions, leading primarily to surface corrosion rather than stable oxides formation [40]. Within the optimal 40–60 V window, the ZnO thickness is controllable and can be tuned by reaction time, yielding approximately 1 µm at 10 s, 5 µm at 30 s, and 10 µm at 60 s (Fig. S10). Therefore, considering the structural integrity, surface morphology, and energy consumption, an oxidation voltage range of 40–60 V and a ZnO layer thickness of 3–5 µm was selected for further investigation.

The introduction of ZnO with porous structure can significantly boost the Zn ion accessibility at the interface. As shown in Figs. 1f and S11, the ZnO-Zn electrodes exhibited an order-of-magnitude increase in capacitance (1.79 vs. 0.11 µF cm^−2^) compared to pristine Zn foil, which is beneficial for Zn deposition. However, it is still challenging for pure ZnO-based interlayers to suppress Zn dendrite formation and water-induced side reactions due to its open structure and rigid nature [23]. To address this, PVA with good ion conductivity (1.7 × 10^−4^ S cm^−1^, Fig. S12) and mechanical flexibility was utilized as the external layer to seal the surface of ZnO-Zn and provide continuous Zn ion transport. As shown in Fig. 1g, PVA can seamlessly infuse into the open pores of the ZnO layer and firmly anchor onto the ZnO-Zn surface during the blade coating process, forming a highly structurally integrated PVA-coated ZnO-Zn hereafter referred to as PZnO-Zn, which features a rigid-to-soft graded interface. The incorporation of PVA also effectively smoothened the surface, resulting in a lower roughness compared with ZnO-Zn (Fig. S13). The total thickness of the ZnO-PVA interfacial layer was controlled to be approximately 5 µm (Fig. 1h), comprising ~ 3-µm ZnO and ~ 2-µm PVA (Fig. S14). Bending tests confirmed the strong adhesion and excellent structural integrity of the hybrid interfacial layer to Zn surface, whereas the PVA layer on PVA-coated Zn (PVA-Zn) foil peeled off immediately after bending (Fig. 1i). It is important to note that the smooth surface of commercial Zn foils often leads to the detachment of polymer-based coating layers. Although surface polishing can increase the surface roughness of Zn foils, the freshly introduced sharp tips and scratches can cause a concentrated electric field distribution, resulting in preferential Zn deposition at these “hot spots”, a phenomenon that will be discussed in the following section. Scratch tests further quantitatively showed that PZnO-Zn exhibited the highest critical load of 3.14 N, compared with that of 1.75 N for ZnO-Zn and 1.11 N for bare Zn (Fig. S15), confirming the enhanced scratch resistance imparted by the hybrid interfacial layer. Therefore, the in situ*-*formed ZnO inner layer with porous structure is crucial for enhancing the adhesion and even distribution of the external PVA layer, ultimately benefiting the structural integrity and stability of the entire ZnO–PVA hybrid interfacial layer.

### Mechanistic Investigation of Zn Deposition Behaviour and Ion Transport Kinetics

**Fig. 2:**
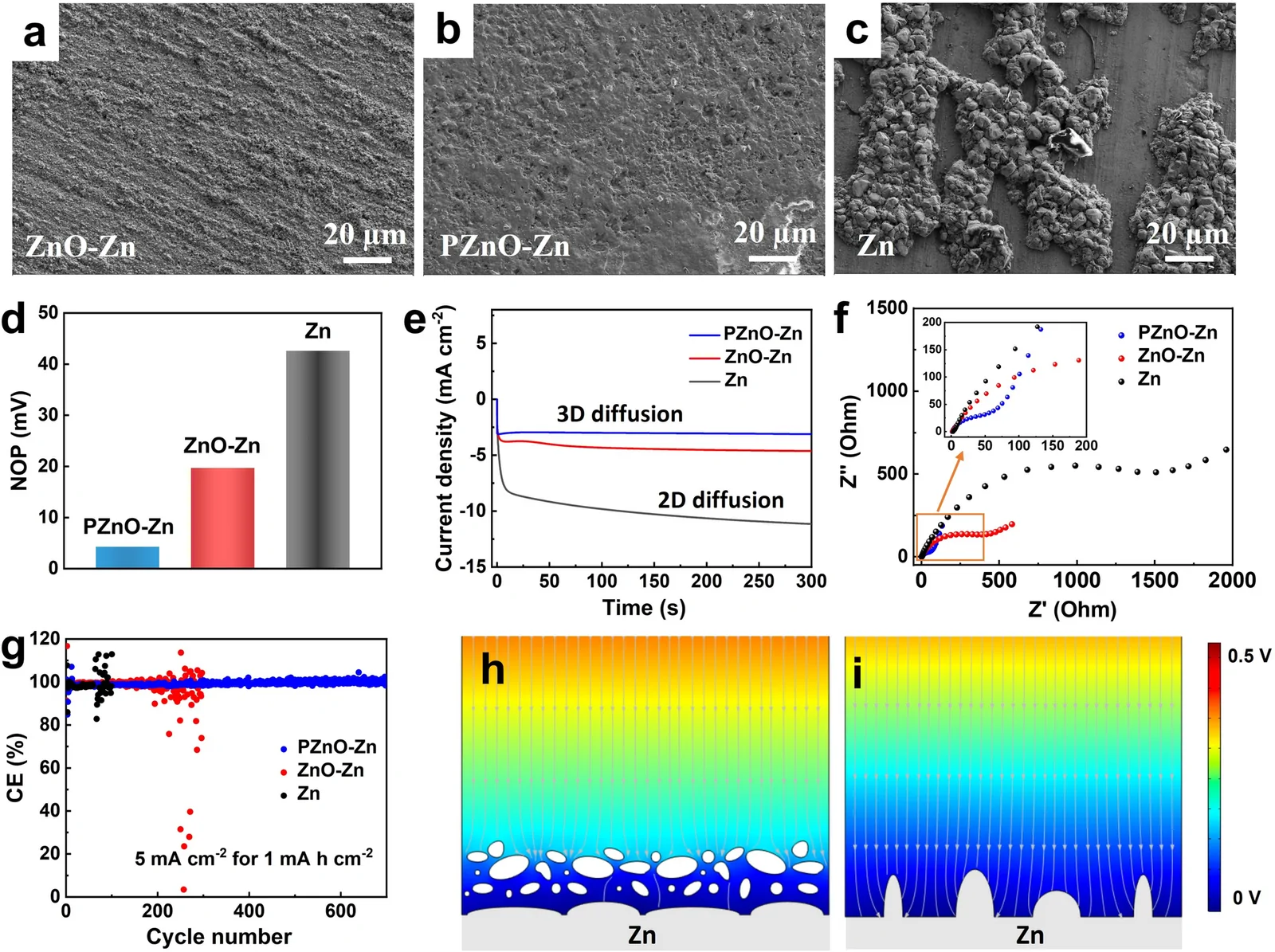
Investigation of Zn deposition behaviour on Zn anodes with different surface properties. **a**–**c** Top-view SEM images of Zn deposition on **a** ZnO-Zn, **b** PZnO-Zn, and **c** Zn at a current density of 0.5 mA cm^−2^ for 0.5 mAh cm^–2^. **d** NOP of PZnO-Zn, ZnO-Zn, and Zn at a current density of 0.1 mA cm^−2^. **e** CA curves of PZnO-Zn, ZnO-Zn, and Zn at a constant voltage of − 150 mV (Zn^2+^/Zn). **f** EIS of PZnO-Zn, ZnO-Zn, and pristine Zn electrodes. **g** CE of PZnO-Zn, ZnO-Zn, and Zn at 5 mA cm^−2^ for 1 mA h cm.^−2^. **h**, **i** Electric field simulation of Zn deposition on **h** PZnO-Zn and **i** pristine Zn (colour represents the electric potential and grey lines with arrows represent the electric field lines)

The Zn deposition behaviour on Zn foils with different surface properties was first examined through ex situ SEM observation. Due to the electron-insulating nature of ZnO and the high interfacial energy between ZnO and Zn, the deposited Zn on ZnO-Zn exhibited an even distribution across the surface, with no cracks or Zn dendrites observed (Fig. 2a). The introduction of the PVA layer further improved the Zn deposition homogeneity, as shown in Fig. 2b. On the contrary, the deposited Zn on pristine Zn foil showed a randomly distributed morphology (Fig. 2c). Zn deposition on Zn foil treated at low oxidation voltage exhibited a preferential Zn deposition, especially at the holes and edges (Fig. S16a, b). This can be attributed to the uneven electric field induced by surface tips and edges, as simulated in Fig. S16c. Similarly, polished Zn foil exhibited preferential deposition along these sandpaper polished grooves (Fig. S17a, b), following a similar mechanism, as simulated in Fig. S17c. However, electric field-induced Zn deposition mechanism demonstrated limited capability for suppressing Zn dendrite formation, as shown in Figs. S16b and S17b, d.

Zn nucleation behaviour was electrochemically studied at a current density of 0.1 mA cm^–2^. As shown in Figs. 2d and S18, the ZnO layer can reduce the Zn nucleation overpotential (NOP) of ZnO-Zn to 19.7 mV compared with 42.6 mV for Zn, which can be attributed to strong interaction between ZnO and Zn ions [30]. As a control, PVA also lowered the NOP to 16.1 mV (Fig. S19a). Remarkably, the integration of PVA into the structural voids of the ZnO layer can significantly reduce the NOP of PZnO-Zn to 4.3 mV, indicating that PVA layer can enhance ion diffusion homogeneity across the interlayer. This uniform nucleation behaviour and reduced NOP trend can be verified by chronoamperogram (CA) test conducted at a constant voltage of − 150 mV (Zn^2+^/Zn). The current density of PZnO-Zn exhibited a stable trend from the beginning of the test (Fig. 2e), reflecting a compact three-dimensional (3D) Zn ion diffusion after nucleation [13]. PVA-Zn showed a longer transition to 3D diffusion and a steeper slope during subsequent Zn growth process (Fig. S19b), compared with PZnO-Zn. ZnO-Zn showed a mild increasing tread in current density, suggesting its limited capability to regulate Zn nucleation behaviour. On the contrary, a rapidly increasing current density was observed for bare Zn, suggesting a rampant two-dimensional (2D) diffusion and uneven Zn dendrite growth process. Electrochemical impedance spectroscopy (EIS) results indicated that PZnO-Zn exhibited the lowest charge transfer resistance of 92.5 Ω, much lower than that of ZnO-Zn, PVA-Zn, and Zn (Figs. 2f and S19c). The improvement in Zn nucleation regulation can be further verified by Coulombic efficiency (CE) test in half cells. As shown in Fig. 2g, PZnO-Zn exhibited the most stable average CE of 99.6% for more than 700 cycles at 5 mA cm^−2^ for 1 mAh cm^−2^, significantly outperforming ZnO-Zn (99.2% for 210 cycles). In contrast, the CE of pristine Zn stabilized for only 60 cycles. Additionally, PVA-Zn, with a similar PVA layer thickness, showed a stable cycling stability for ~ 300 cycles (Fig. S19d). The CE comparison among PZnO-Zn, PVA-Zn, ZnO-Zn, and pristine Zn underscores the synergistic effects of ZnO and PVA in enhancing the reversibility of Zn deposition.

To better understand the mechanism why uniform Zn deposition can be achieved on PZnO-Zn foil, an electric field simulation was conducted. In contrast to the simulated electrical field distribution of polished Zn and Zn foil anodized at a low voltage between 10 and 20 V (Figs. S16c and S17c), the introduction of the porous ZnO-based interfacial layer dramatically improved the uniformity of the local electric field within the interfacial layer, as shown in Fig. 2h. The even electric field can subsequently uniform the ion distribution and Zn deposition. On the contrary, the randomly distributed Zn dendrite deposited on pristine Zn foil resulted in severely localized electric field at the tips and protrusions (Fig. 2i), leading to preferential Zn ion movement toward these “hot spots” and the formation of Zn dendrites.

**Fig. 3:**
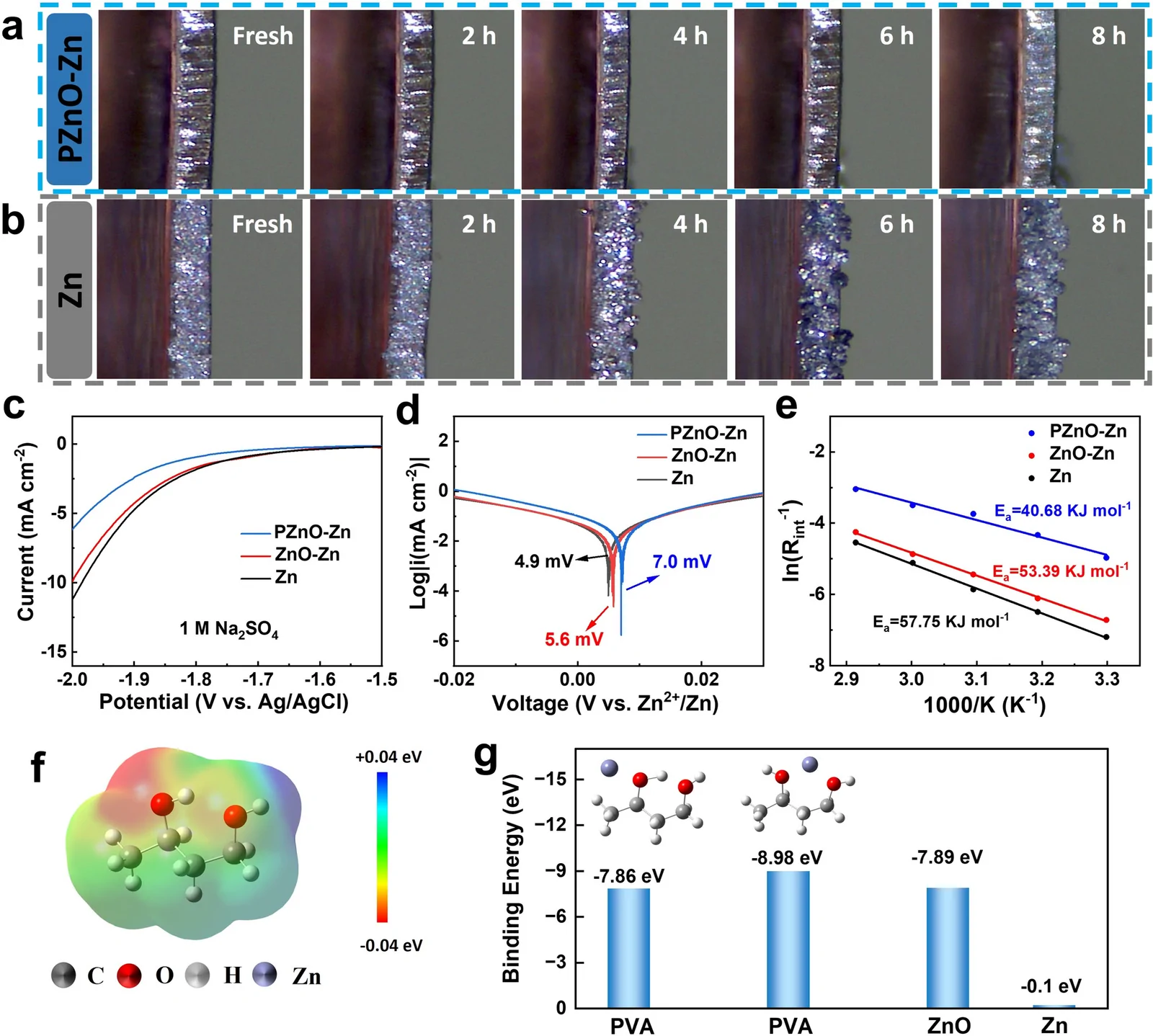
Analysis and mechanism study of the hybrid interfacial layer. **a**, **b **In situ optical observation of the Zn plating/stripping on **a** PZnO-Zn and **b** Zn at a current density of 5 mA cm^−2^ for 1 mAh cm^−2^. **c** LSV curves of PZnO-Zn, ZnO-Zn, and Zn electrodes in 1 M Na_2_SO_4_ at a scan rate of 5 mV s^−1^. **d** Linear polarization and **e** Arrhenius curves of PZnO-Zn, ZnO-Zn, and Zn electrodes. **f** ESP of PVA. **g** DFT calculation of the binding energy between Zn ion and PVA and the comparison with the values of ZnO and Zn derived from previous studies [42], insets showing the corresponding molecular models for the calculations

In situ optical observation of all electrodes was conducted at 5 mA cm^−2^ for 1 mAh cm^−2^ to verify the morphological change of the electrodes. The cross-sectional images revealed that the morphology of PZnO-Zn remained unchanged after the test (Fig. 3a). On the contrary, pristine Zn exhibited rapid morphological decay and dendrite formation after approximately 4 h (Fig. 3b). ZnO-Zn also displayed a mild decaying process during the test (Fig. S20), suggesting that pure ZnO interlayer alone cannot effectively protect Zn from corrosion. To explore the reason for the improved surface stability and CE of the hybrid interlayer, the polarization behaviours of the electrodes were measured by linear sweep voltammetry (LSV) in 1 M Na_2_SO_4_ electrolyte to accurately study the HER. As shown in Fig. 3c, ZnO-Zn exhibited reduced HER compared to pristine Zn anode, indicating improved HER suppression provided by the ZnO layer. More importantly, PZnO-Zn demonstrated a dramatically reduced HER current density, highlighting the superior HER inhibition provided by the hybrid interfacial layer. As a control, polished Zn showed the highest current density (Fig. S21), indicating that surface roughness and sharp tips of polished Zn foil can also exacerbate the corrosion of electrode. The hybrid layer’s ability to suppress side reactions was further verified by the linear polarization tests (Fig. 3d). Compared to ZnO-Zn (5.6 mV vs. Zn^2+^/Zn) and Zn (4.9 mV vs. Zn^2+^/Zn), PZnO-Zn showed the most positive potential of 7.0 mV (vs. Zn^2+^/Zn), indicating significantly improved corrosion resistance [13].

To gain deeper insights into the mechanism behind the reduced side reactions, the activation energy (*E*_a_) was employed to evaluate the Zn ion transport kinetics within the hybrid interfacial layer [36]. The *E*_a_ can be determined by measuring the temperature-dependent interfacial transfer resistance (R_int_) of Zn ion derived from the EIS, according to the Arrhenius equation below:

5 $$\frac{1}{{R_{{{\mathrm{int}}}} }} = A {\text{exp }}\left( { - \frac{{E_{{\mathrm{a}}} }}{RT}} \right)$$

where *E*_a_ is the activation energy,* A* is the pre-exponential factor, *T* is the absolute temperature, and *R* is the gas constant. As shown in Figs. 3e, S22 and S23, both the ZnO and PVA layer can reduce the activation energy of ZnO-Zn to 53.39 and 50.71 kJ mol^−1^, respectively, compared to 57.75 kJ mol^−1^ for pristine Zn electrode. Notably, the ZnO/PVA hybrid interfacial layer further lowered the activation energy to 40.68 kJ mol^−1^, indicating a facilitated Zn ion desolvation process at the electrode interface [41]. To elucidate such synergistic interactions responsible for this enhancement, the electrostatic potential map (ESP) was calculated based on the chemical structure of PVA. As shown in Fig. 3f, PVA exhibits higher local electronegativity near oxygen atoms, supporting the preferential binding affinity for positively charged Zn ions. DFT calculations further support this interaction, revealing that the binding energy between Zn ion and the oxygen of PVA is − 7.86 eV on the of top oxygen atom and − 8.98 eV in the middle of two adjacent oxygen atoms (Fig. 3g and insets). These values indicate that the Zn–O interaction between PVA and Zn ion is slightly stronger than that between ZnO and Zn ions, and both are significantly higher than that between Zn (002) and Zn ion, as summarized in Fig. 3g [42]. Therefore, the effective desolvation capability of the hybrid ZnO/PVA interfacial layer can be attributed to its strong interaction with Zn ions, occurring through a two-step process: Zn ions first interact with the PVA top layer, and subsequently with both PVA and ZnO within the ZnO scaffold, as schematically illustrated in Fig. S24.

### Electrochemical Performance of PZnO-Zn Anode

**Fig. 4:**
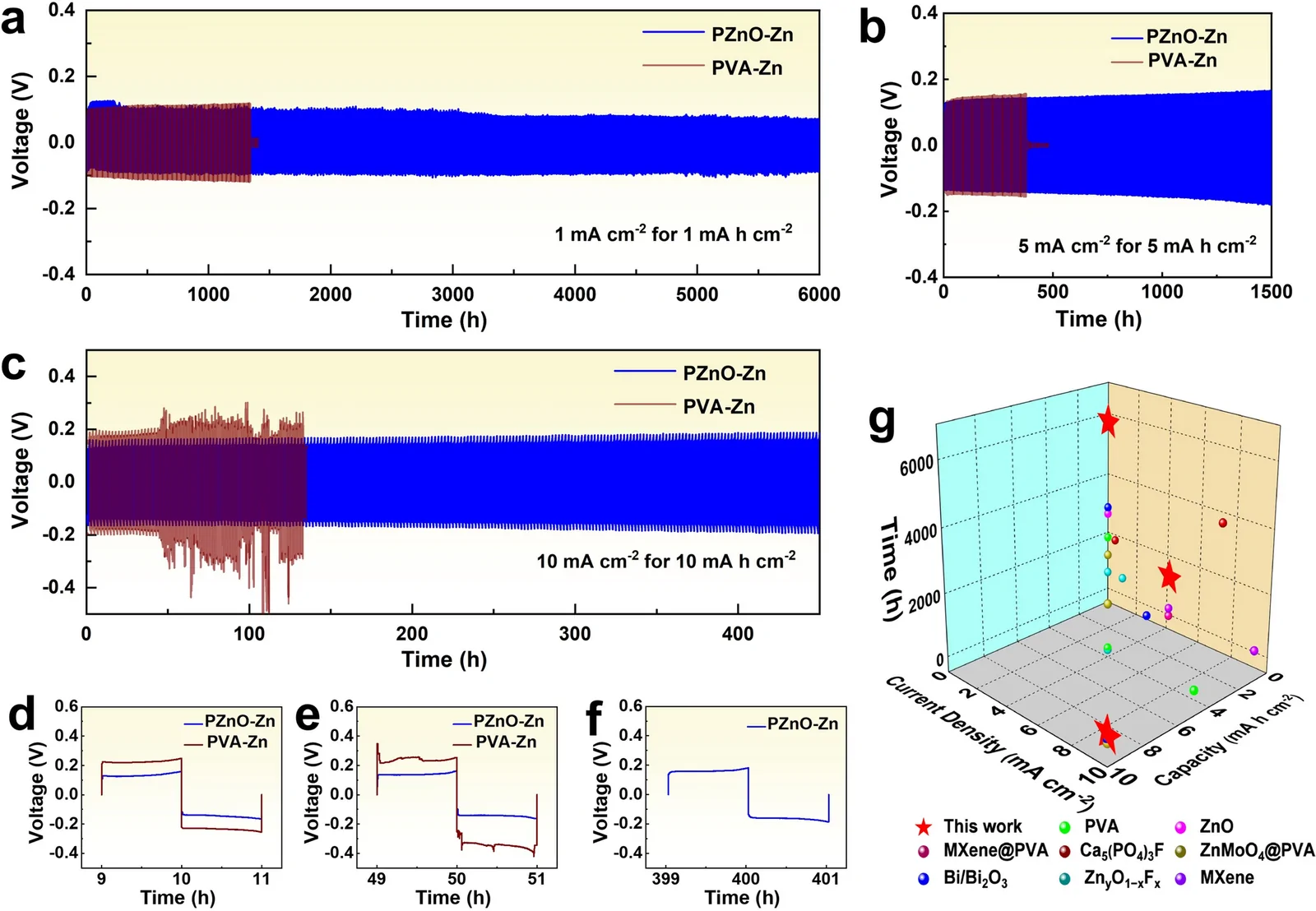
Electrochemical performance of PZnO-Zn in symmetrical cells. **a**–**c** Cycling stability of PZnO-Zn and PVA-Zn at **a** 1 mA cm^−2^ for 1 mA h cm^−2^, **b** 5 mA cm^−2^ for 5 mAh cm^−2^, and **c** 10 mA cm^−2^ for 10 mAh cm^−2^. **d**–**f** Enlarged cycling profiles of **c** at different cycling time. **g** Comparison of the cycling lifespan of PZnO-Zn with other reported works

Symmetric cells were assembled to test the cycling stability of the Zn anodes in a coin cell configuration. As shown in Fig. S25, the rate performance demonstrated that PZnO-Zn exhibited superior cycling stability, with a higher exchange current density (4.1 mA cm^−2^) compared with PVA-Zn (1.9 mA cm^−2^), suggesting that the reduction Zn^2+^ ion at the interface is more efficient and better regulated [43]. In contrast, ZnO-Zn and bare Zn showed unstable voltage profiles at high current densities (Fig. S26). The hybrid interlayer enabled exceptional Zn plating/stripping reversibility in PZnO-Zn for more than 6000 h at 1 mA cm^−2^ for 1 mAh cm^−2^, significantly outperforming PVA-Zn. As a control (Fig. 4a), ZnO-Zn showed superior cycling stability than Zn under the test condition (Fig. S27), confirming the beneficial role of ZnO-based interfacial layer in enhancing Zn anode durability. However, ZnO-Zn exhibited voltage fluctuation after 450 h, suggesting that single-component ZnO-based interlayer with loose structure was insufficient to suppress Zn dendrite formation or side reactions. Post-cycling morphology after 100 h was examined by ex situ SEM. Bare Zn exhibited pronounced surface roughening with widespread pits and voids (Fig. S28a, b). ZnO-Zn also displayed dendritic protrusions on the surface of the ZnO layer (Fig. S28c, d). PVA-Zn showed a clear suppression of dendrite formation with no obvious Zn dendrite observed (Fig. S28e, f). By contrast, the hybrid PZnO-Zn presented the most uniform surface with the PVA overlayer retained in the ZnO scaffold (Fig. S28g, h), indicating enhanced interfacial robustness of the hybrid interfacial layer and more homogeneous Zn deposition. XRD patterns revealed additional reflections in the low-angle region for cycled Zn, ZnO-Zn, and PVA-Zn electrodes (Fig. S29), which can be assigned to Zn_x_(OH)_y_(CF_3_SO_3_)_z_·nH_2_O [44]. In contrast, such reflections were absent for PZnO-Zn, further confirming that the interlayer effectively suppresses the formation of by-products. The performance comparison of PZnO-Zn, ZnO-Zn and PVA-Zn revealed the limitations of using either an inorganic ZnO layer or an organic PVA layer alone to improve the cycling stability of the Zn anodes.

When the current density was increased into 5 mA cm^−2^ for 1 mAh cm^−2^, PZnO-Zn showed a high cycling stability of more than 1,700 h, outperforming PVA-Zn, ZnO-Zn, and Zn (Figs. S30 and S31). Even at an increased areal capacity of 5 mAh cm^−2^, PZnO-Zn achieved a long cycling stability of 1,500 h, surpassing PVA-Zn (Fig. 4b). Furthermore, when cycled at a high current density of 10 mA cm^−2^ with a high areal capacity of 10 mAh cm^−2^, PZnO-Zn maintained a long cycling stability of approximately 450 h (Fig. 4c). Enlarged plating and stripping profiles showed that PZnO-Zn remained a stable voltage polarization throughout the test (Fig. 4d–f). In contrast, PVA-Zn showed poor cycling stability with significant voltage fluctuations appearing after approximately 50 h (Fig. 4e), indicating a mild internal short-circuit caused by unstable PVA-Zn surface. Even under a more challenging electrolyte of 2 M ZnSO_4_ [45], PZnO-Zn exhibits robust cycling stability of over 1000 h at 5 mA cm^−2^ for 5 mAh cm^−2^ and 350 h at 10 mA cm^−2^ for 10 mAh cm^−2^ (Fig. S32). The cycling lifespan of PZnO-Zn electrodes at these testing conditions surpasses that of most reported Zn anodes with single or hybrid interface protection strategies under similar testing conditions, as summarized in Fig. 4g and Table S1.

### Practical Application of PZnO-Zn Anode in Rechargeable Zinc-Iodine Batteries

**Fig. 5:**
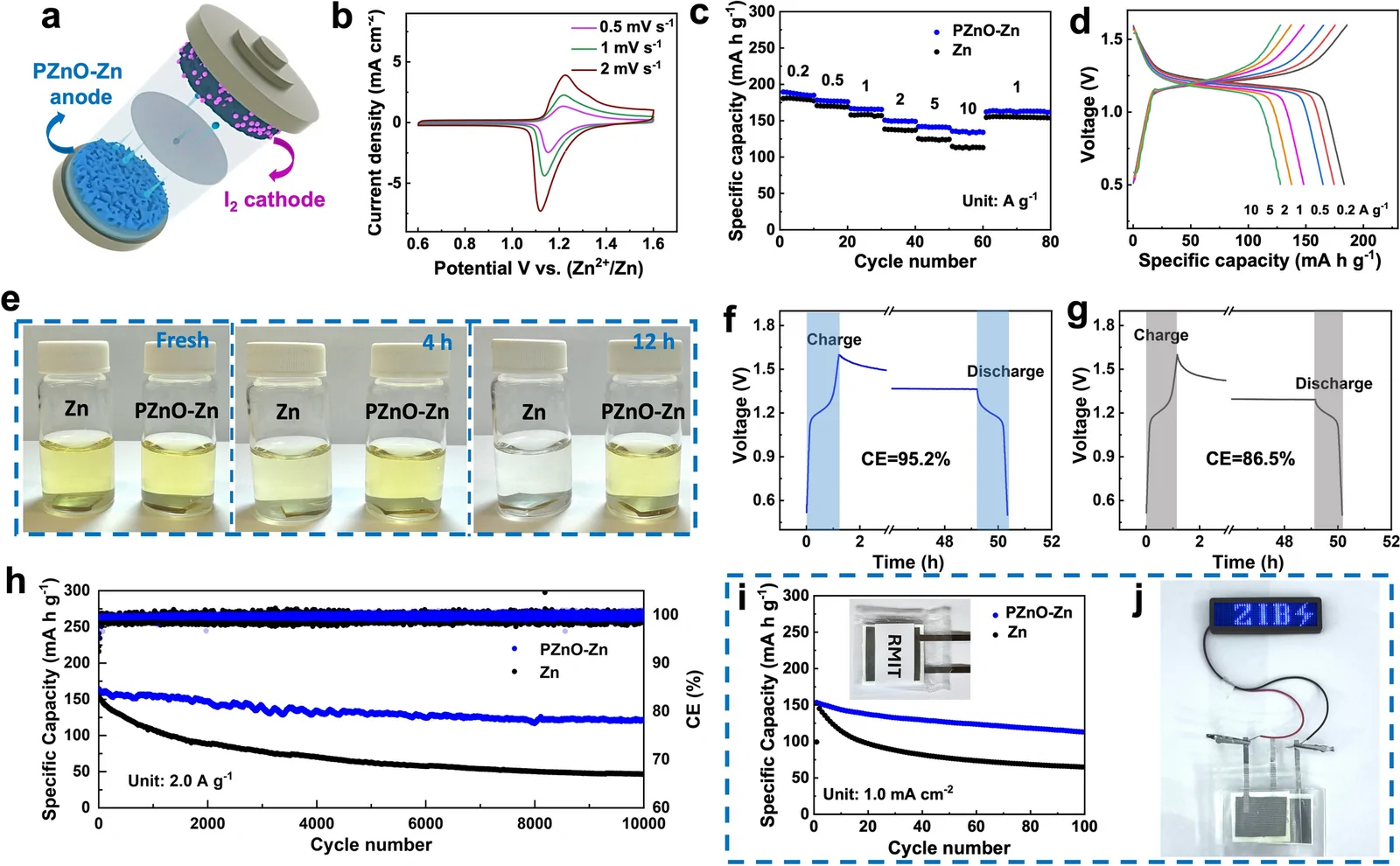
Practical application of PZnO-Zn in Zn||I_2_ full cells. **a** Schematic of PZnO-Zn||I_2_ batteries. **b** CV curves of PZnO-Zn||I_2_ batteries at different scan rates. **c** Rate performance of PZnO-Zn||I_2_ and Zn||I_2_ full cells. **d** Charge and discharge profiles of PZnO-Zn||I_2_ cell at different current densities. **e** Visual observation of the immersion experiment of PZnO-Zn and Zn in I_3_^−^ solutions. **f, g** Self-discharge tests of **f** PZnO-Zn||I_2_ and **g** Zn||I_2_ full cells. **h** Long cycling tests of PZnO-Zn||I_2_ and Zn||I_2_ full cells at 2.0 A g^−1^. **i** The cycling performance of pouch batteries at 1.0 mA cm^−2^. **j** Digital images of a LED panel lighted by two PZnO-Zn||I_2_ pouch cells connected in series

Rechargeable zinc-iodine (Zn-I_2_) batteries present a promising opportunity for large-scale and safe energy storage system [46, 47]. However, directly using Zn as anodes poses a challenge due to the corrosion caused by polyiodine intermediates [48, 49]. The practical application of PZnO-Zn was evaluated in Zn-I_2_ full cells, as illustrated in Fig. 5a. Cyclic voltammetry (CV) curves of the full cells displayed characteristic redox peaks of cathodes at different scan rates, indicating good reversibility of the I_2_ cathode (Fig. 5b) [50]. The rate performance showed that the Zn-I_2_ full cells with PZnO-Zn anodes (PZnO-Zn||I_2_) achieved an initial specific capacity of 189.5 mAh g^−1^ at 0.2 A g^−1^, with good capacity retention at increased current densities. This performance was notably superior to that of Zn-I_2_ full cells with pristine Zn anodes (Zn||I_2_), as shown in Fig. 5c. Furthermore, the stable charge and discharge plateaus of PZnO-Zn||I_2_ full cells at these current densities verified the favourable polyiodide conversion reaction (Fig. 5d). The designed hybrid interface can effectively prevent the corrosion of PZnO-Zn anode by polyiodine. As shown in Fig. 5e, the yellow colour of polyiodine solution gradually faded after approximately 4 h when pristine Zn foil was immersed. The solution colour became completely transparent after ageing for 12 h, indicating the complete consumption of polyiodine due to oxidation. SEM images of Zn foils after the ageing test confirmed the surface corrosion (Fig. S33a, b). However, PZnO-Zn anode retained its original yellow colour for more than 12 h with no obvious surface changes (Fig. S33c, d), indicating effective corrosion resistance provided by the hybrid interfacial layer. As a control, the ZnO layer of ZnO-Zn showed limited oxidation resistance against polyiodide due to its open structure (Fig. S34). A self-discharge test further verified the effectiveness of the hybrid interfacial layer in suppressing the side reactions. After 48 h of rest, PZnO-Zn can still retain a high CE of 95.2% (Fig. 5f), significantly higher than the 86.5% for pristine Zn (Fig. 5g). Benefitting from the hybrid interfacial layer with improved cycling stability and corrosion resistance against HER and polyiodide corrosion, the PZnO-Zn||I_2_ achieved a stable cycling stability for more than 5000 cycles at 0.5 A g^−1^ (Fig. S35). On the contrary, Zn||I_2_ cells showed rapid capacity decay and fluctuating CE. When the current density was increased to 2.0 A g^−1^, PZnO-Zn||I_2_ can deliver a specific capacity of approximately 160 mAh g^−1^ and maintain a stable CE over 10,000 cycles, exhibiting a low capacity decay of ~ 0.02‰ per cycle (Fig. 5h). For comparison, PVA-Zn||I_2_ also showed better cycling performance than pure Zn, with a decay rate of ~ 0.028‰ per cycle versus ~ 0.07‰ for pure Zn (Fig. S36). The application of PZnO-Zn anodes was further assessed in a pouch cell configuration. As shown in Fig. 5i, PZnO-Zn||I_2_ exhibited improved capacity retention than that with pristine Zn anode, showing enhanced corrosion resistance. When connected in series, the PZnO-Zn||I_2_ pouch cells successfully powered a LED panel (Fig. 5j), demonstrating the feasibility of its practical application. The compatibility of the PZnO-Zn anode was further demonstrated in the Zn-MnO_2_ full cell (Fig. S37), which delivered significantly enhanced cycling stability and sustained capacity retention compared with the Zn counterpart, highlighting the broad applicability and practical potential of the designed hybrid interfacial layer for advanced aqueous Zn-based batteries.

## Conclusion

In this study, we develop a hybrid interfacial layer with rigid-to-soft structure, consisting of an in situ*-*formed inorganic ZnO inner layer and organic ion-conducting PVA external layer for Zn anode protection. A fast and scalable liquid plasma-assisted oxidation process was designed and systematically investigated for the preparation of ZnO-Zn. The porous ZnO inner layer with high interfacial energy and mechanical modulus not only effectively regulates Zn nucleation behaviour and suppresses Zn dendrite formation, but also enhances the adhesion and structure integrity of the external PVA layer. The ion-conductive PVA layer can further homogenize the ion distribution and inhibit the side reactions. Computational and experimental results demonstrated that the ZnO-PVA hybrid interfacial layer with strong interaction with Zn ions can synergistically homogenize the electrical field and facilitates the desolvation of water from Zn ions, resulting in enhanced surface stability and corrosion resistance. As a result, PZnO-Zn can achieve a long cycling stability of more than 6000 h at 1 mA cm^−2^ for 1 mAh cm^−2^ and 450 h at 10 mA cm^−2^ for 10 mAh cm^−2^ in symmetric cells. Additionally, the hybrid interlayer can effectively prevent Zn corrosion by polyiodine when used as anodes in Zn-I_2_ full cells, enabling a stable cycling and minimal capacity decay (~ 0.02‰ per cycle) for more than 10,000 cycles at 2.0 A g^−1^.

## Supplementary Information

Below is the link to the electronic supplementary material.

Supplementary file1 (DOCX 6054 KB)
