# Supplementary material for: A Rigid–Soft Graded Organic–Inorganic Interlayer for Durable and Corrosion-Resistant Zinc Anodes
Source: Nanomicro Lett. 2026 Jan 5;18:175. doi: 10.1007/s40820-025-02020-8 (PMC12765758; doi:10.1007/s40820-025-02020-8)
Supplement: Supplementary file 1 — Supplementary file1 (DOCX 6054 KB) [file 40820_2025_2020_MOESM1_ESM.docx]

Supporting Information for

**A Rigid-Soft Graded Organic-Inorganic Interlayer for Durable and Corrosion-Resistant Zinc Anodes**

Zhiyu Wang^1^, Junlun Cao^1^, Zixuan Yang^2^, Jianli Cheng^3^, Dan Liu^1*^, Weiwei Lei^1*^

^1^ School of Science, RMIT University, Melbourne, VIC 3000, Australia

^2^ Institute for Frontier Materials, Deakin University, Waurn Ponds Campus, Locked Bag 20000, Victoria 3220, Australia

^3^ School of Optoelectronic Science and Engineering, University of Electronic Science and Technology of China, Chengdu 611731, P. R. China

^*^Corresponding authors. E-mail: [dan.liu@rmit.edu.au](mailto:dan.liu@rmit.edu.au) (Dan Liu); [weiwei.lei@rmit.edu.au](mailto:weiwei.lei@rmit.edu.au) (Weiwei Lei)

**Supplementary Figures**


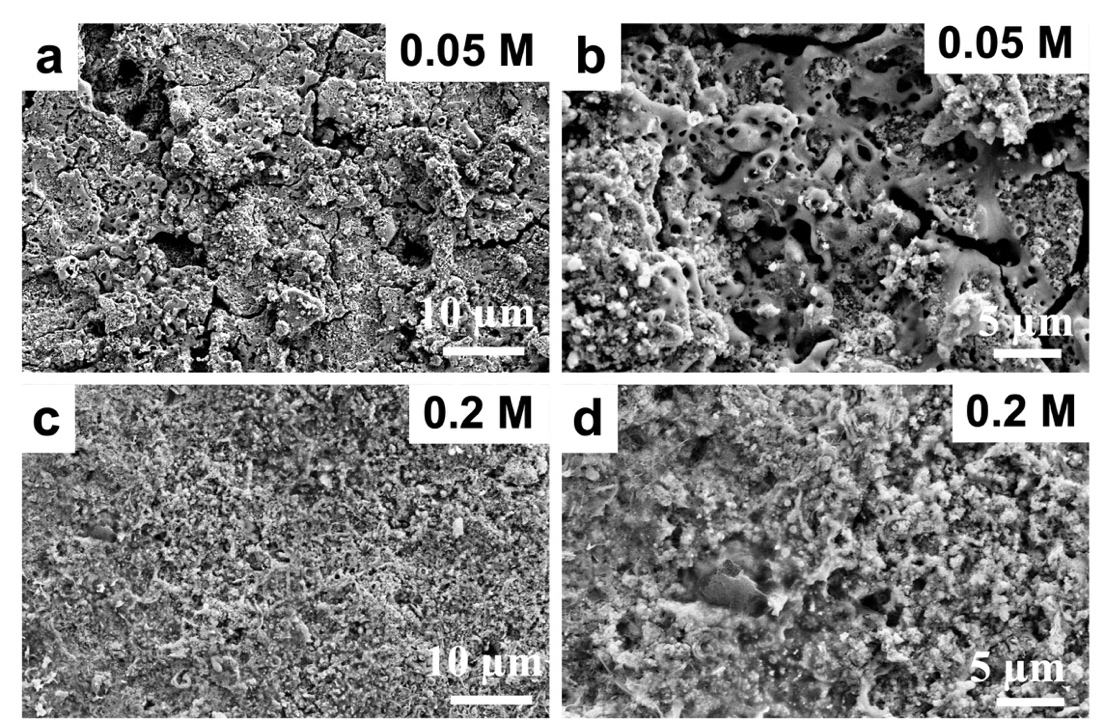


**Fig. S1** SEM of Zn foil treated in KOH solution with a concentration of (**a, b**) 0.05 M and (**c, d**) 0.2 M

Supporting note: The breakdown voltage, corresponding to the initiation of arc discharge that prevents further voltage elevation, decreases with increasing KOH concentration, from ~300 V for 0.05 M KOH to ~110 V for 0.2 M KOH.


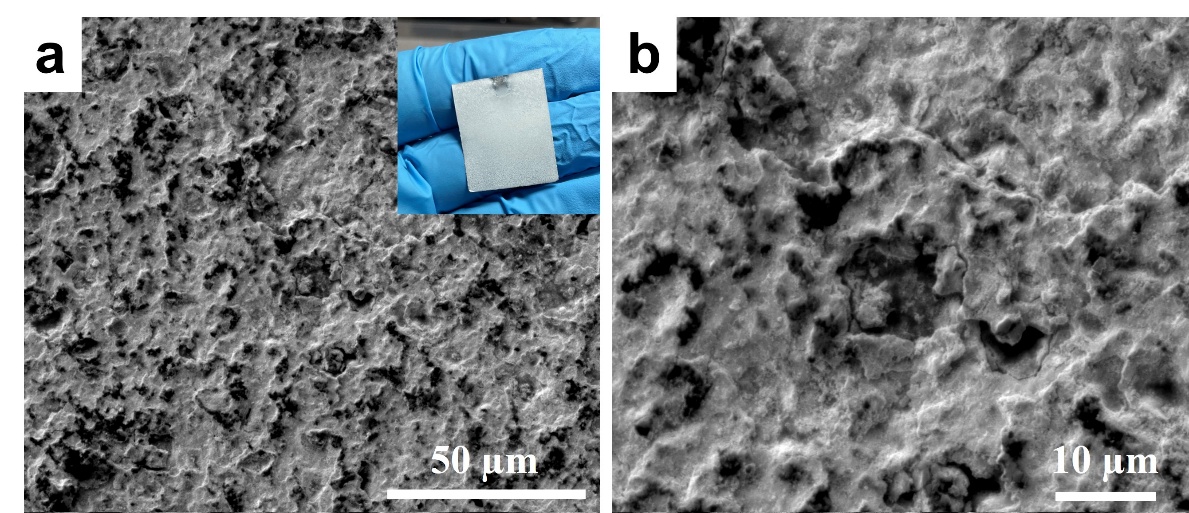


**Fig. S2** SEM of Zn foil treated in 0.1 M NaOH solution at a voltage between 40-60 V (Inset is the digital image of anodized Zn foil)


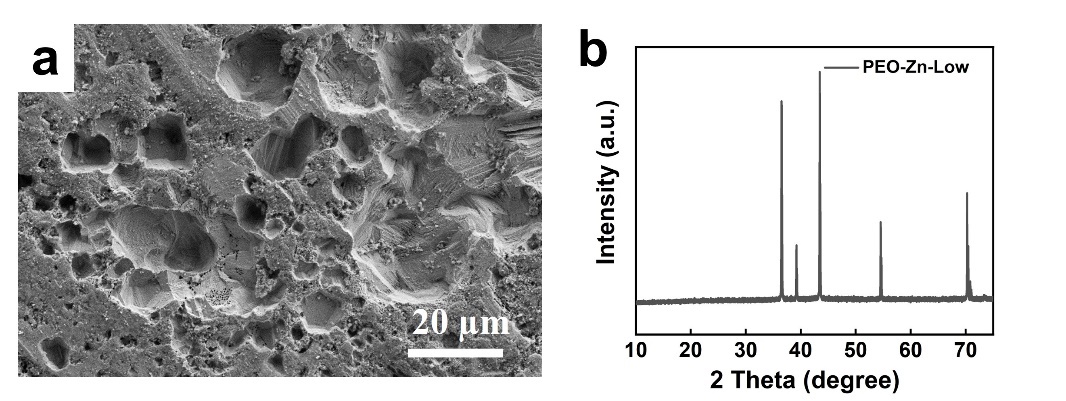


**Fig. S3** (**a**) SEM image and the (**b**) XRD pattern of Zn foil treated in a voltage range between 10-20 V


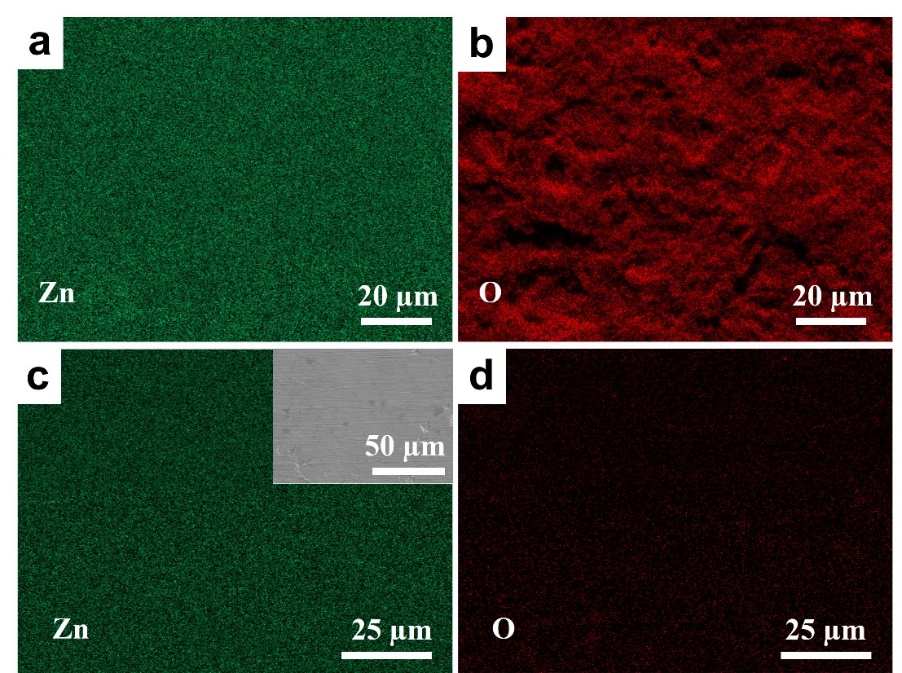


**Fig. S4** EDX mappings of ZnO-Zn show the distribution of (**a**) Zn and (**b**) O elements. EDX mappings of pristine Zn shows the distribution of (**c**) Zn and (**d**) O elements, (inset is the SEM image of pristine of Zn foil)


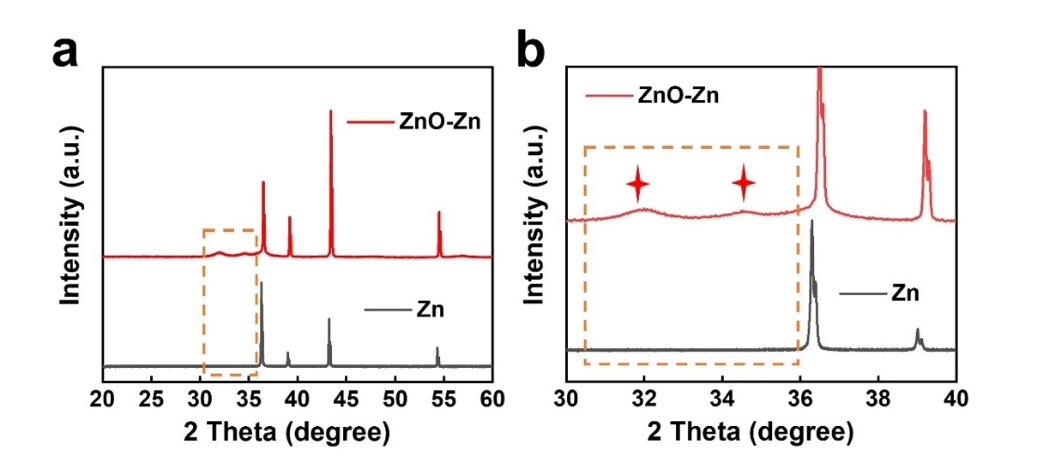


**Fig. S5** XRD pattern (**a**) of pristine Zn (black) and ZnO-Zn anodized between 40-60 V, and (**b**) the corresponding magnified view of (a)


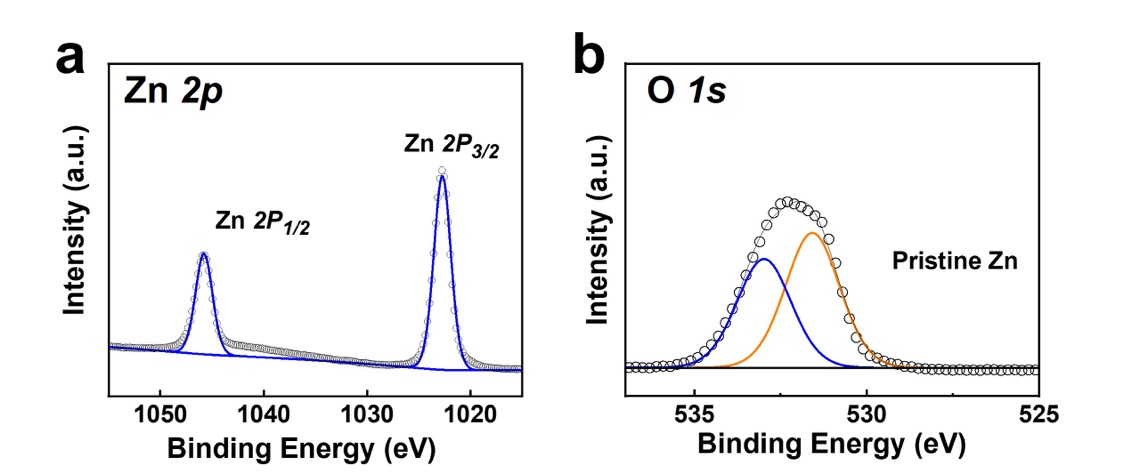


**Fig. S6** High-resolution (**a**) Zn 2p and (**b**) O 1s XPS spectra of pristine Zn foil


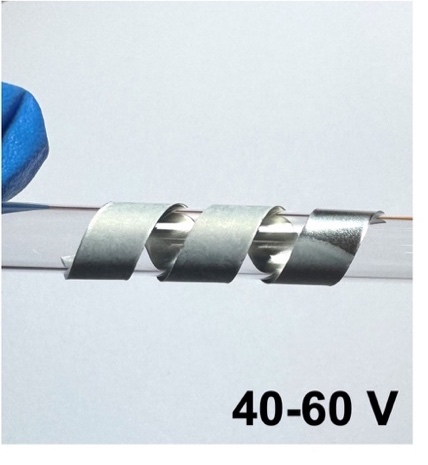


**Fig. S7** Digital images of ZnO-Zn anodized at 40-60 V wrapped on a glass pipette


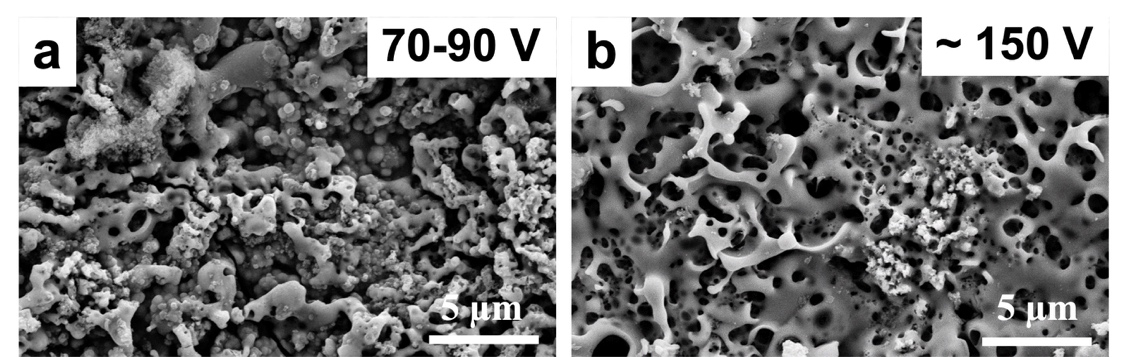


**Fig. S8** Top-view SEM images of pristine Zn foils anodized in a voltage range between (**a**) 70-90 V and (**b**) at ~150 V


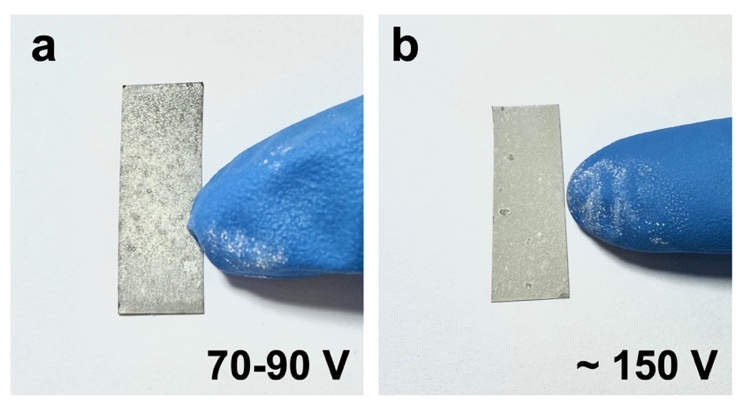


**Fig. S9** Digital images of ZnO-Zn anodized between (**a**) 70-90 V, and (**b**) at approximately 150 V, both showing some white powder sticking to the glove when touched with a finger


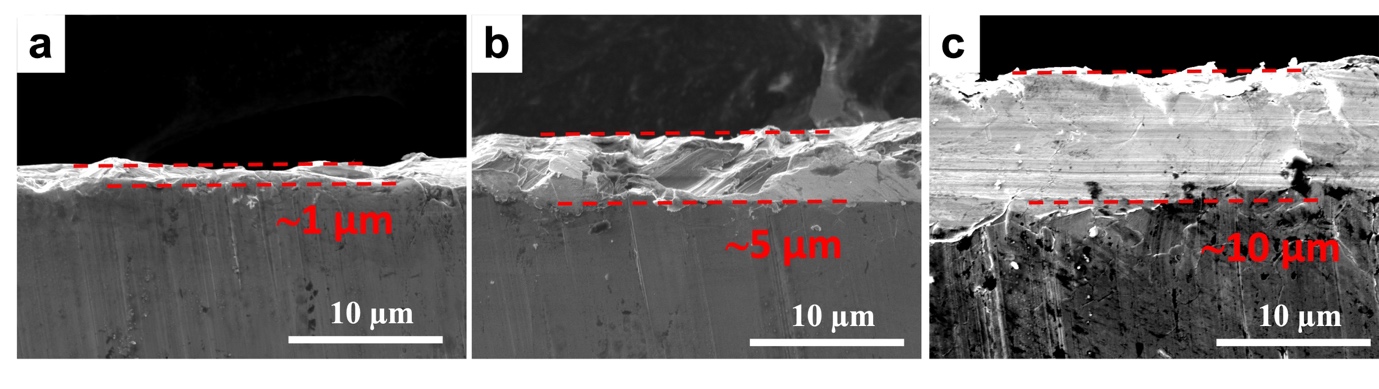


**Fig. S10** Cross section images of ZnO-Zn anodized between 40-60 V for (**a**) 10 s, (**b**) 30 s, and (**c**) 60 s


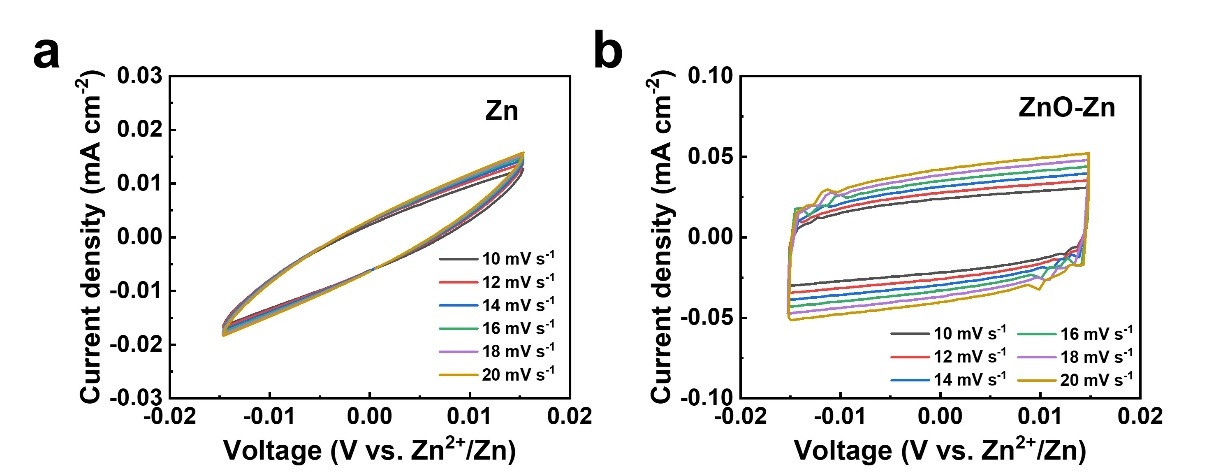


**Fig. S11** CV curves of (**a**) pristine Zn and (**b**) Zn anodized between 40-60 V at different scan rates

**Fig. S12** EIS of cells assembled with two spacers (black) and assembled with one stainless-steel spacer and one spacer coated with a PVA layer (brown)

Supporting notes: The ionic conductivity of PVA layer can be estimated based on the following equation:

$$\sigma= \frac{L}{RS}$$

where R is the resistance, S is the area (1.767 cm^–2^), and L is the thickness of the PVA layer (2 µm in this work). The impedance of this blank cell represents the background resistance coming from the coin cell cases, spacer, separator, and electrolyte. This background resistance was subtracted from that of the PVA-coated cell, yielding an ionic conductivity of 1.7 × 10^–4^ S cm^–1^ for the PVA film under the tested condition.

**
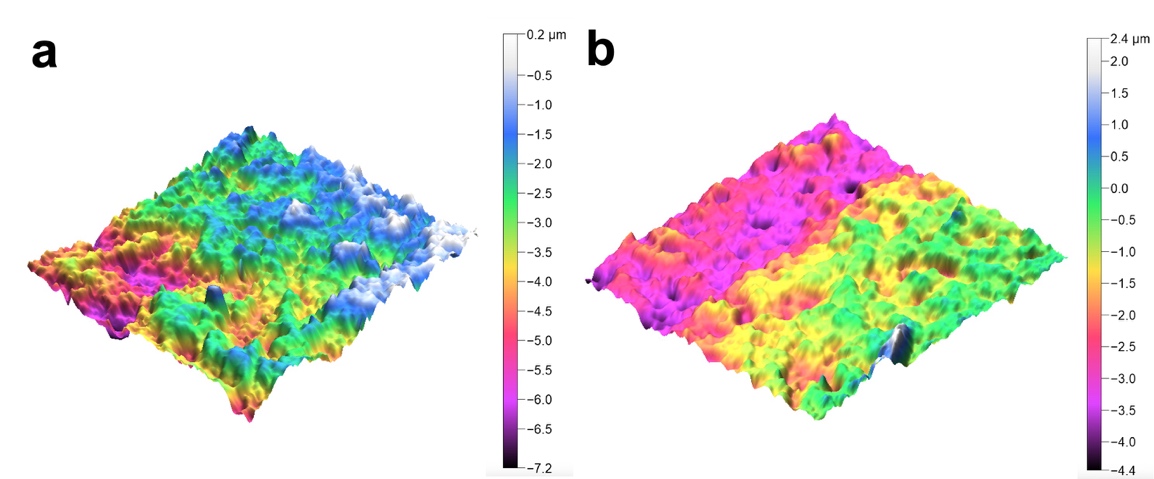
**

**Fig. S13** Surface roughness of (**a**) ZnO-Zn and (**b**) PZnO-Zn


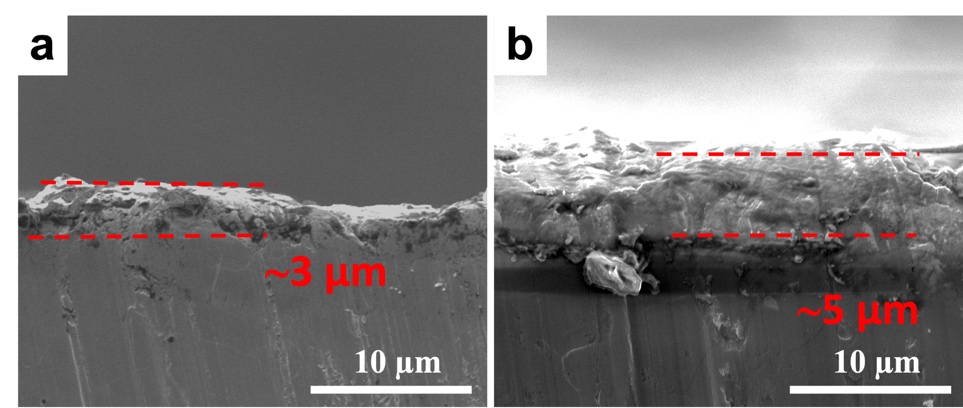


**Fig. S14** Cross section SEM images of (**a**) ZnO-Zn and (**b**) PZnO-Zn


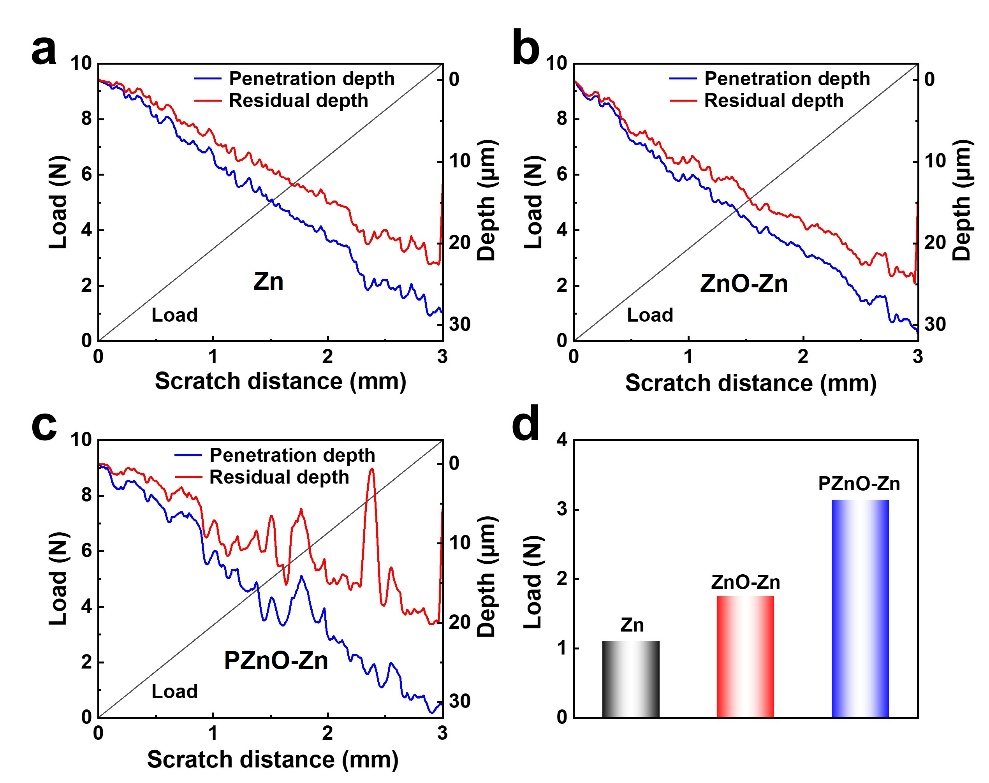


**Fig. S15** Scratch test curves of (**a**) Zn, (**b**) ZnO-Zn, and (**c**) PZnO-Zn. (**d**) Summary of the critical load of the three samples


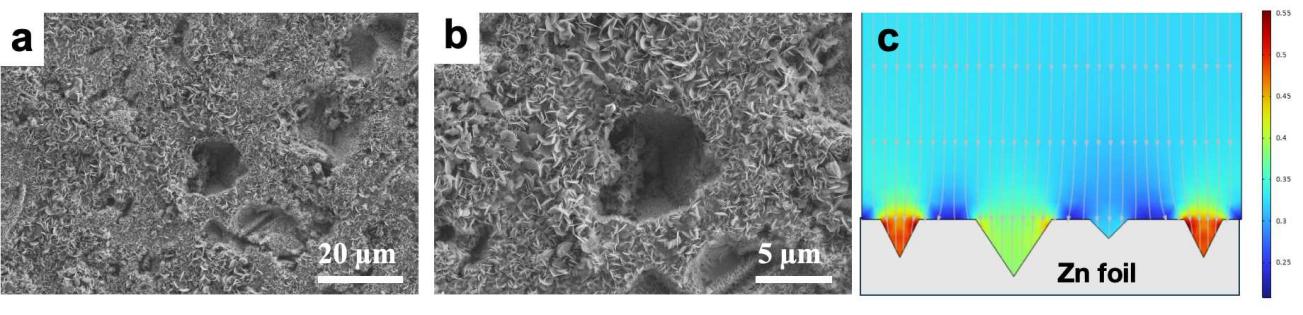


**Fig. S16** (**a** and **b**) SEM images of Zn deposition on Zn foil treated at low voltage between 10-20 V. (**c**) Simulation of the electric field distribution of Zn foil treated at low oxidation voltage of 10-20 V

Supporting note: The simulated electric field distribution of Zn foil treated at low voltage between 10-20 V showed that holes and valleys on Zn foil can dramatically influence the local distribution of the electric field, leading to preferential Zn deposition at these holes. However, the formation of Zn dendrite can still be observed (b).


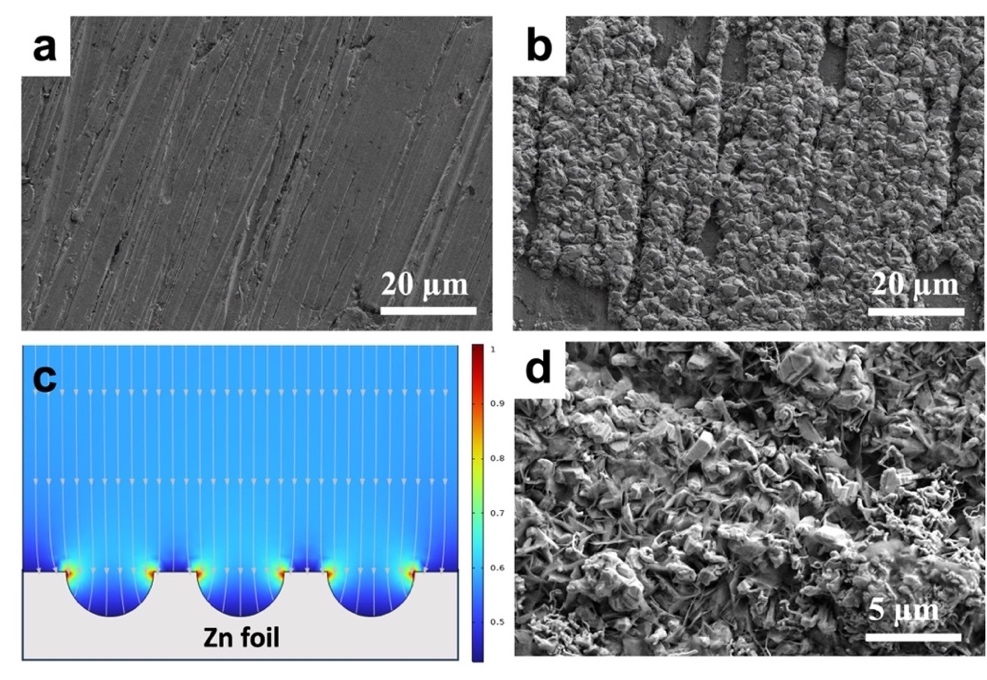


**Fig. S17** SEM images of (**a**) polished Zn foil and (**b**) Zn deposition on polished Zn. (**c**) Simulation of the electric field distribution of polished Zn foil treated at low oxidation voltage. (**d**) Enlarged SEM image of (b)

Supporting note: The simulated electric field distribution of polished Zn showed that the surface roughness of the polished Zn can dramatically influence the local distribution of the electric field especially at the curves and edges of the polished Zn, leading to preferential Zn deposition at these “hot spots” (c). However, this cannot effectively eliminate the formation of Zn dendrite (d).


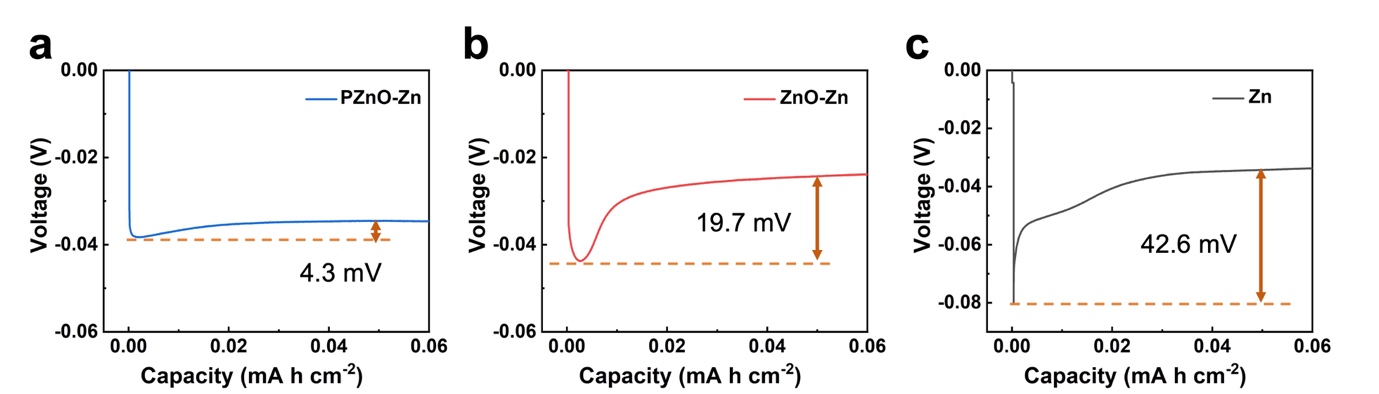


**Fig. S18** Zn deposition profiles of (**a**) PZnO-Zn, (**b**) ZnO-Zn, and (**c**) Zn at a current density of 0.1 mA cm^–2^ in symmetric cells


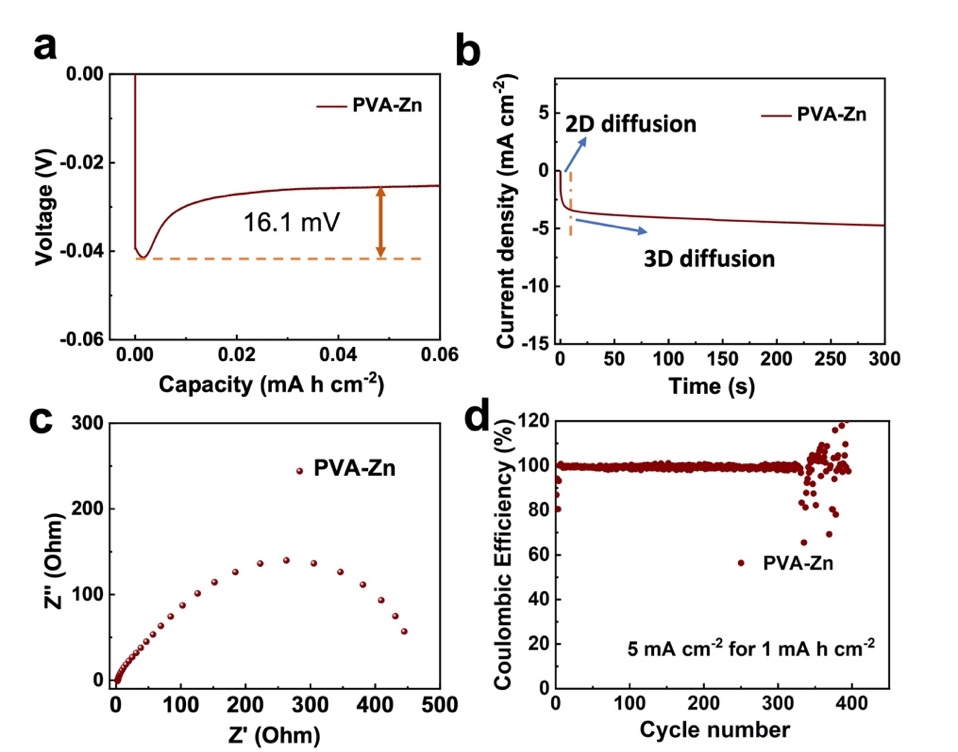


**Fig. S19** NOP (**a**), CV curve (**b**), EIS (**c**), and CE (**d**) of PVA-Zn electrodes as a control


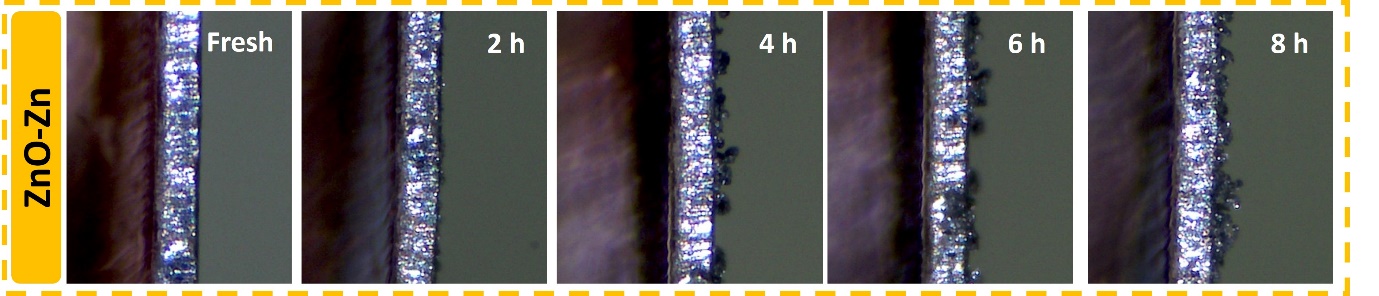


**Fig. S20** *In-situ* optical observation of the Zn plating/stripping on ZnO-Zn at a current density of 5 mA cm^–2^ for 1 mA h cm^–2^

**Fig. S21** LSV of polished Zn foil in 1 M Na_2_SO_4_


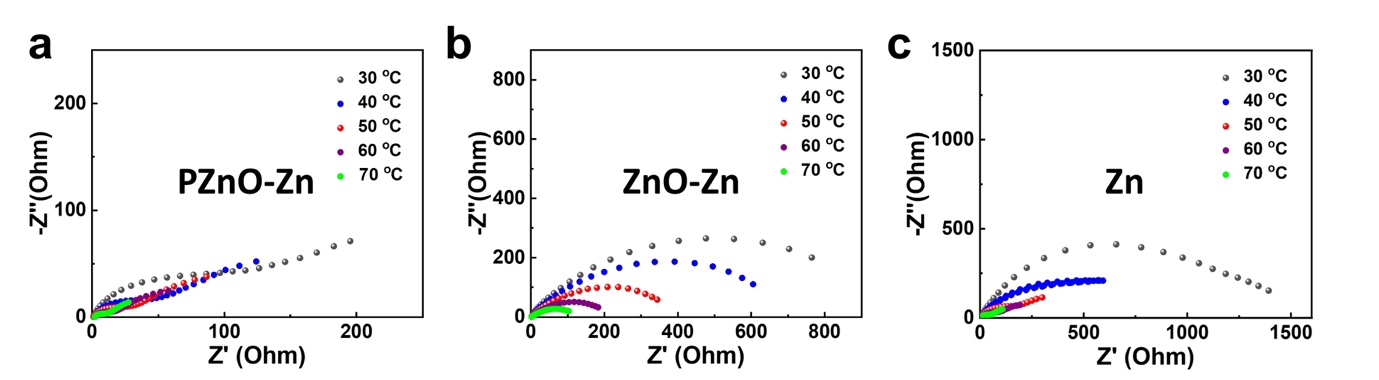


**Fig. S22** EIS tests of symmetric cells using (**a**) PZnO-Zn, (**b**) ZnO-Zn, and (**c**) Zn electrodes at different temperatures


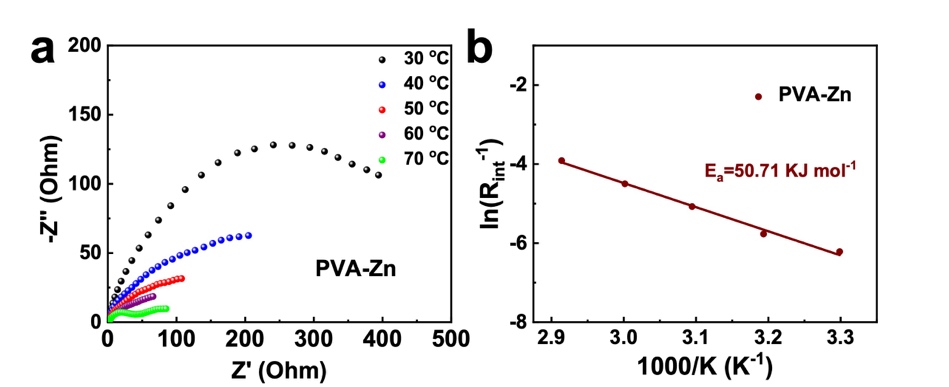


**Fig. S23** (**a**) EIS tests of symmetric cells using PVA-Zn at different temperatures. (**b**) Arrhenius curves of PVA-Zn electrodes


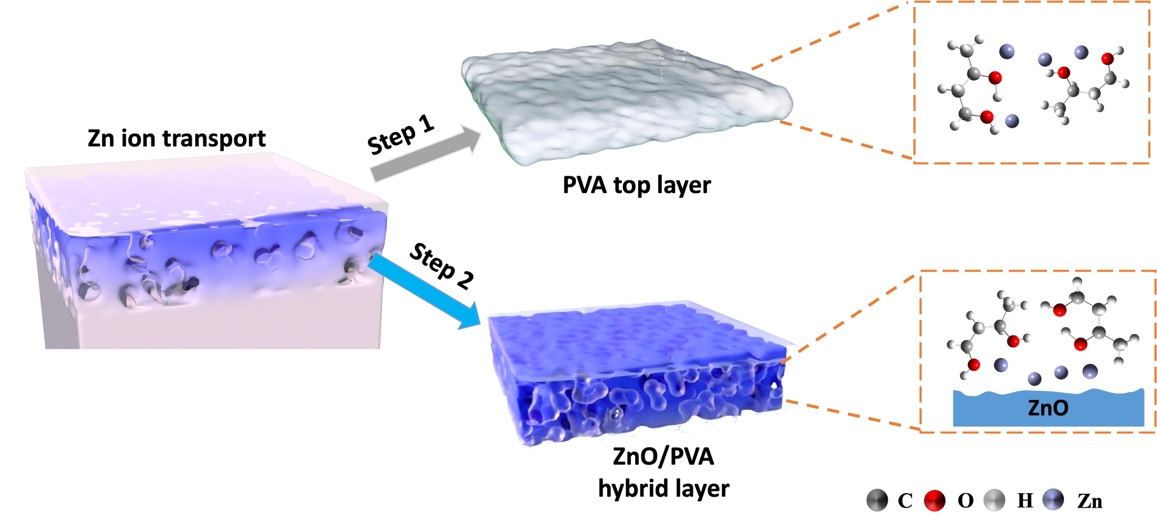


**Fig. S24** Schematic illustration of the two-step interaction between Zn ions and the ZnO/PVA hybrid interfacial layer


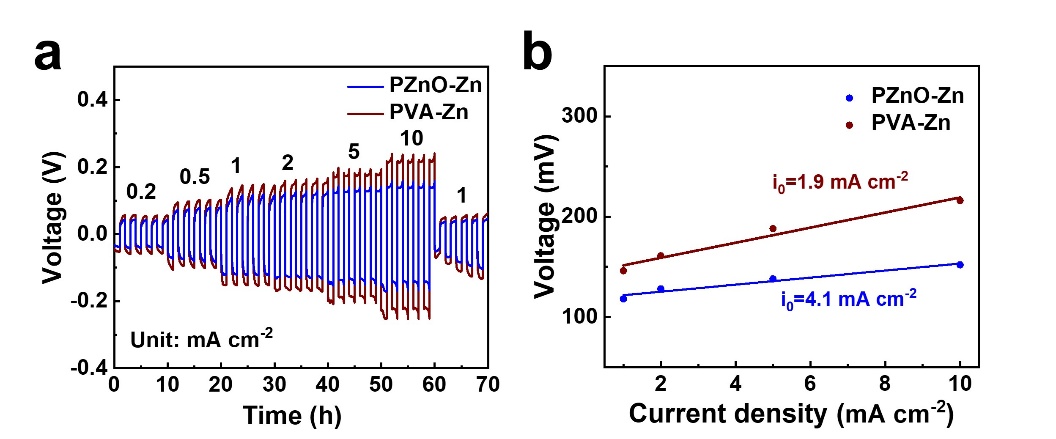


**Fig. S25** (**a**) Rate performance of symmetric PZnO-Zn and PVA-Zn cells at different current densities and (**b**) corresponding exchange current density

**Fig. S26** Rate performance of symmetric ZnO-Zn and Zn cells at different current densities


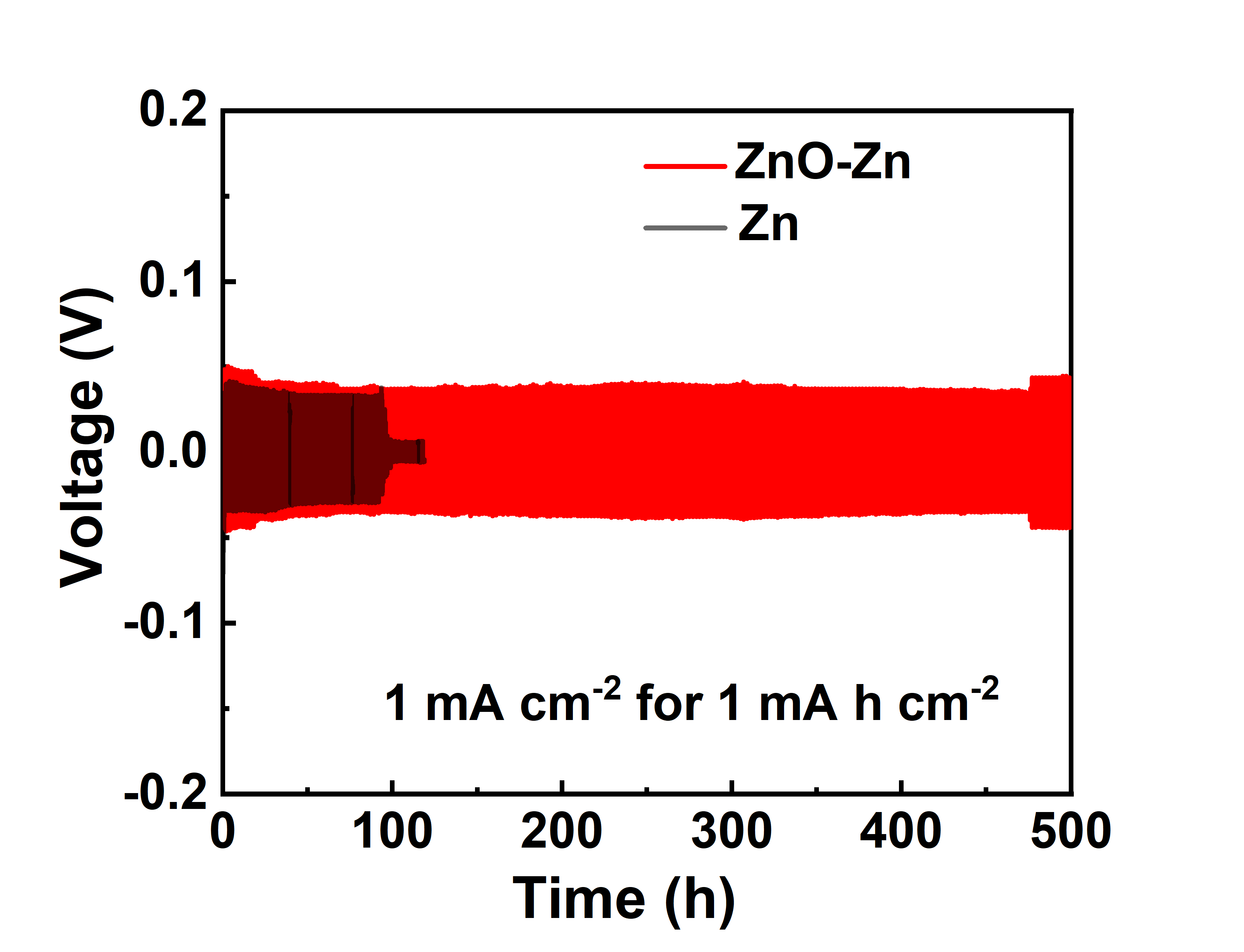


**Fig. S27** Cycling stability test of symmetric ZnO-Zn and Zn cells at 1 mA cm^–2^ for 1 mA h cm^–2^


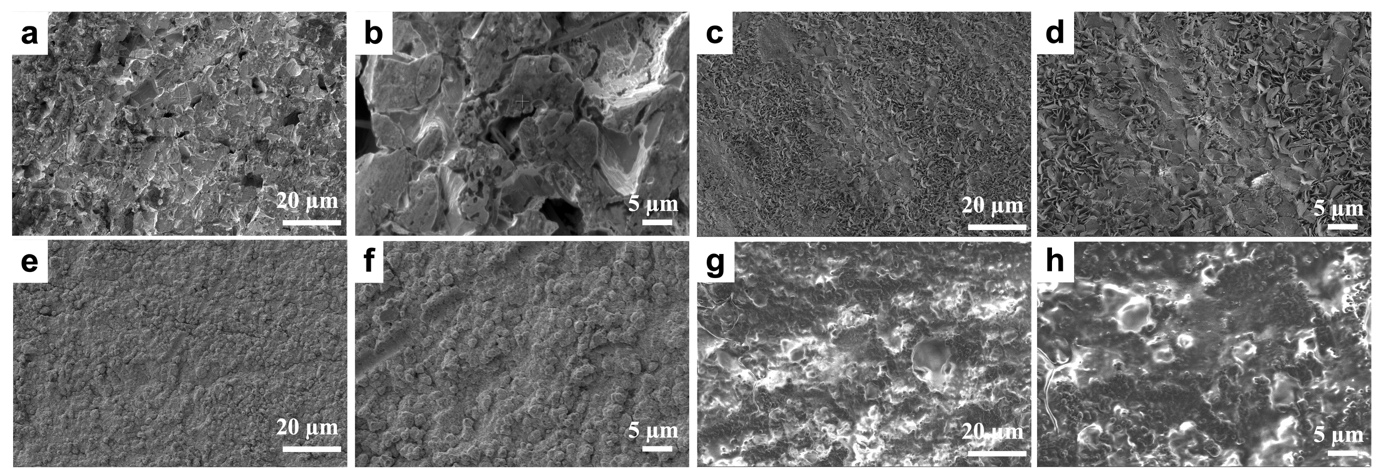


**Fig. S28** SEM images of (**a, b**) Zn, (**c, d**) ZnO-Zn, (**e, f**) PVA-Zn, and (**g, h**) PZnO-Zn after cycling for 100 h at 1 mA cm^–2^ for 1 mA h cm^–2^

Supporting note: For PVA-Zn, a residual PVA film was still discernible on the surface of PVA-Zn electrode upon cell disassembly. However, during the subsequent washing and rinsing step, the PVA film of PVA-Zn electrode detached, and no remaining PVA layer can be observed in the SEM images (Figs. S28e, f).


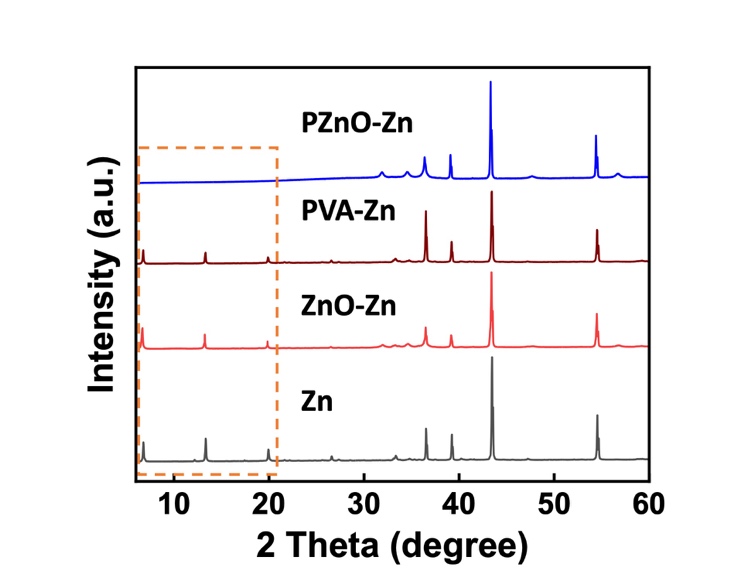


**Fig. S29** XRD profiles of Zn, ZnO-Zn, PVA-Zn, and PZnO-Zn after cycling for 100 h at 1 mA cm^–2^ for 1 mA h cm^–2^. The reflections highlighted by the orange dashed box are attributed to the formation of the Zn_x_(OH)_y_(CF_3_SO_3_)_z_·nH_2_O by-product.


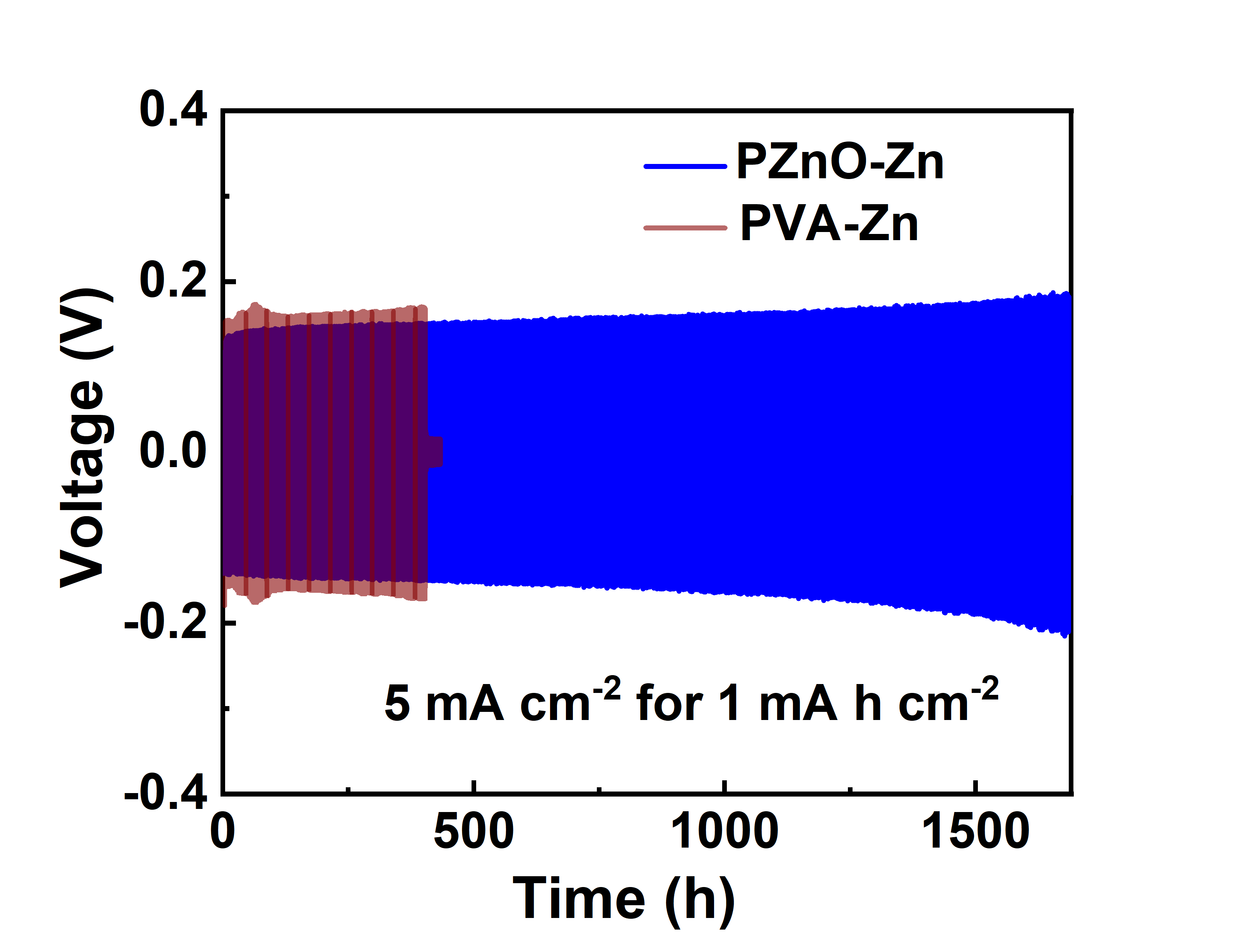


**Fig. S30** Cycling stability test of symmetric PZnO-Zn and PVA-Zn cells at 5 mA cm^–2^ for 1 mA h cm^–2^


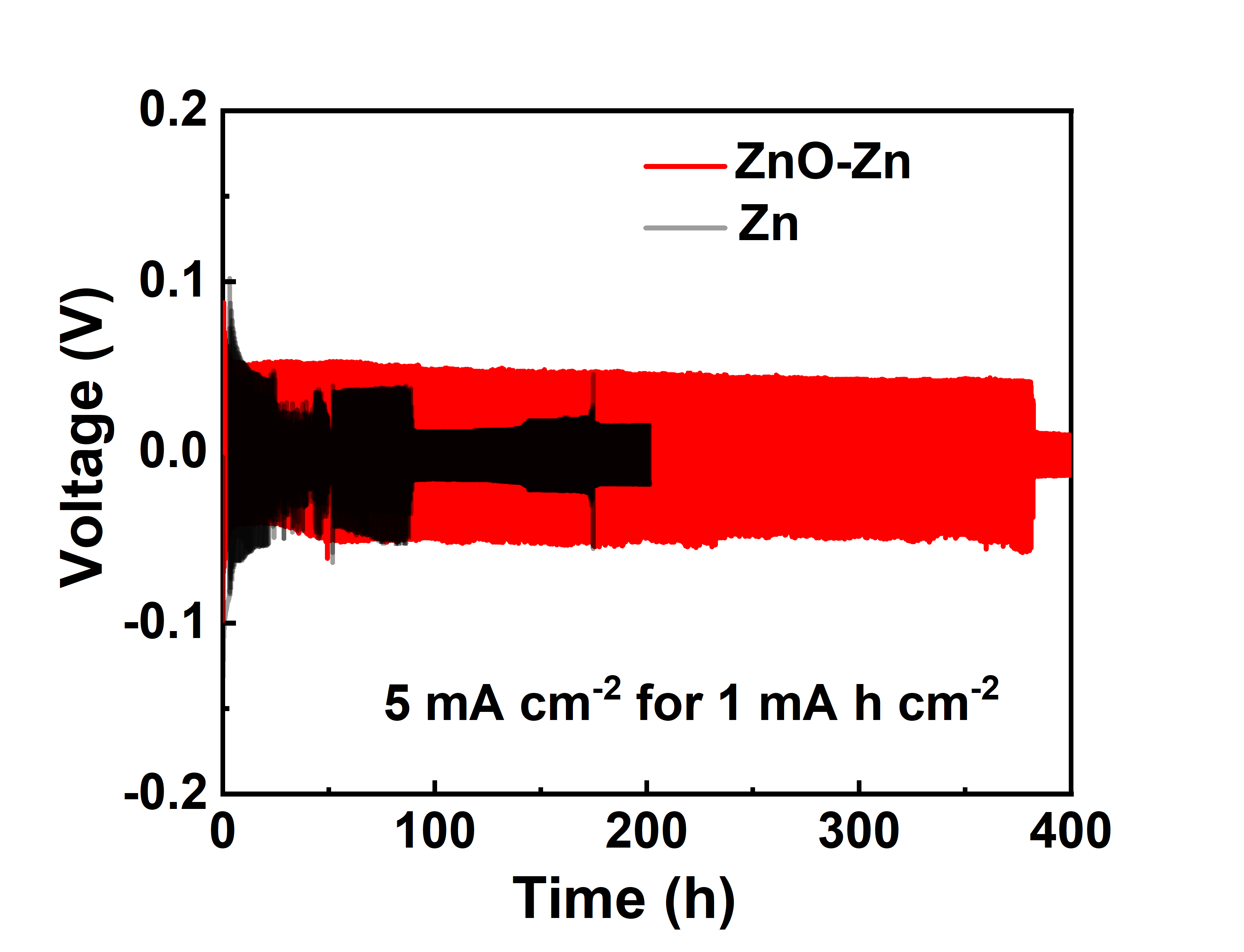


**Fig. S31** Cycling stability test of symmetric ZnO-Zn and Zn cells at 5 mA cm^–2^ for 1 mA h cm^–2^


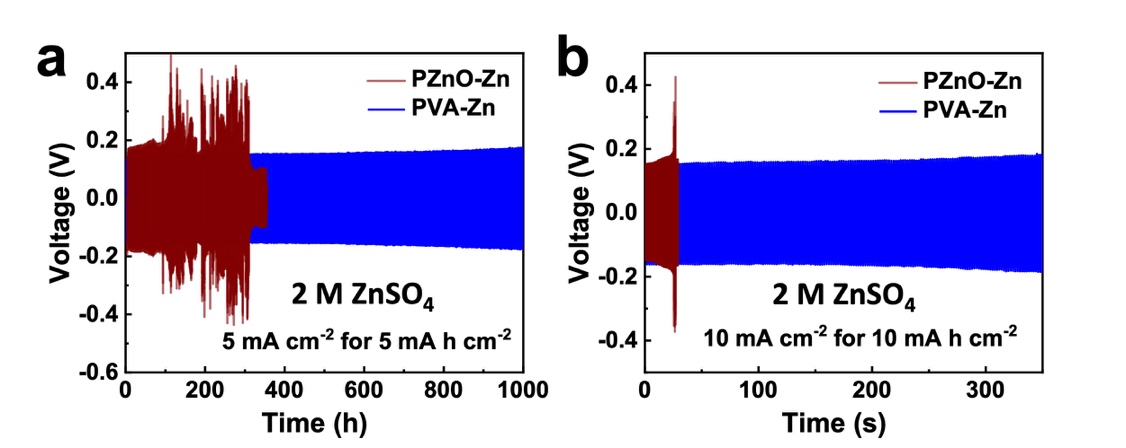


**Fig. S32** Cycling stability tests of symmetric PZnO-Zn and PVA-Zn cells at (**a**) 5 mA cm^–2^ for 5 mA h cm^–2^ and (**b**) 10 mA cm^–2^ for 10 mA h cm^–2^ in 2 M ZnSO_4_ electrolyte


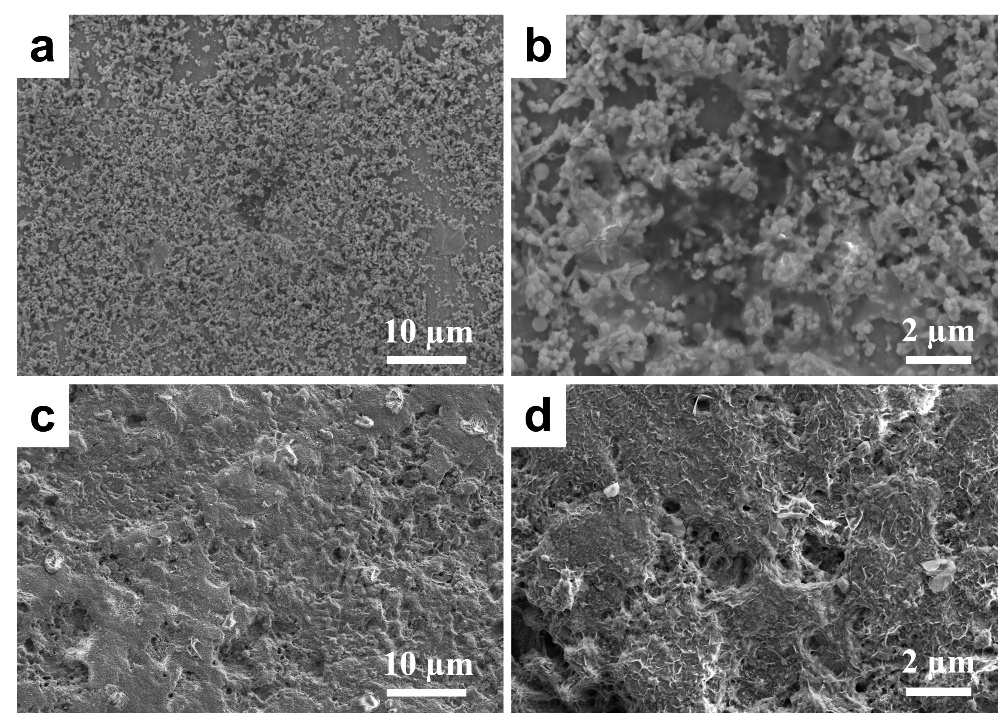


**Fig. S33** SEM images of (**a, b**) Zn anode and (**c, d**) PZnO-Zn after immersion in polyiodine for 12 h


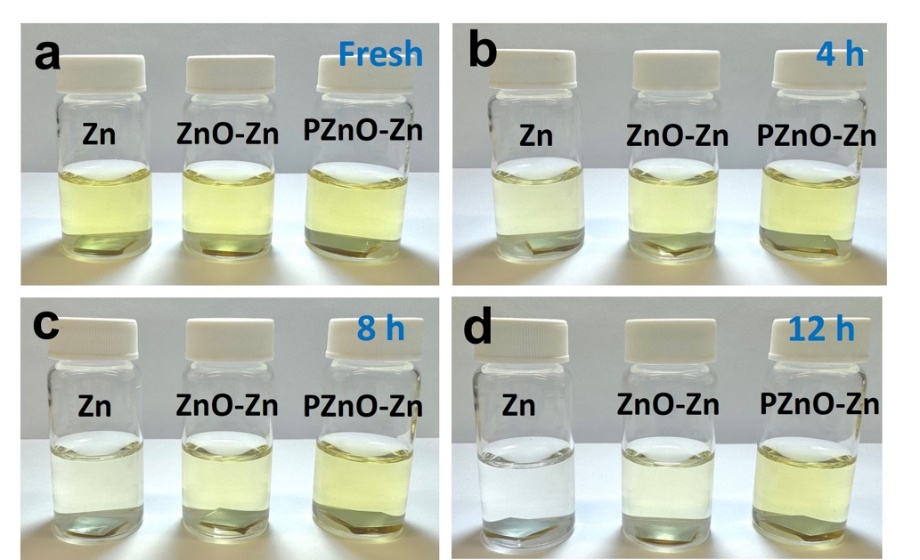


**Fig. S34** Digital images of PZnO-Zn, ZnO-Zn, and Zn in polyiodine solution for 12 h


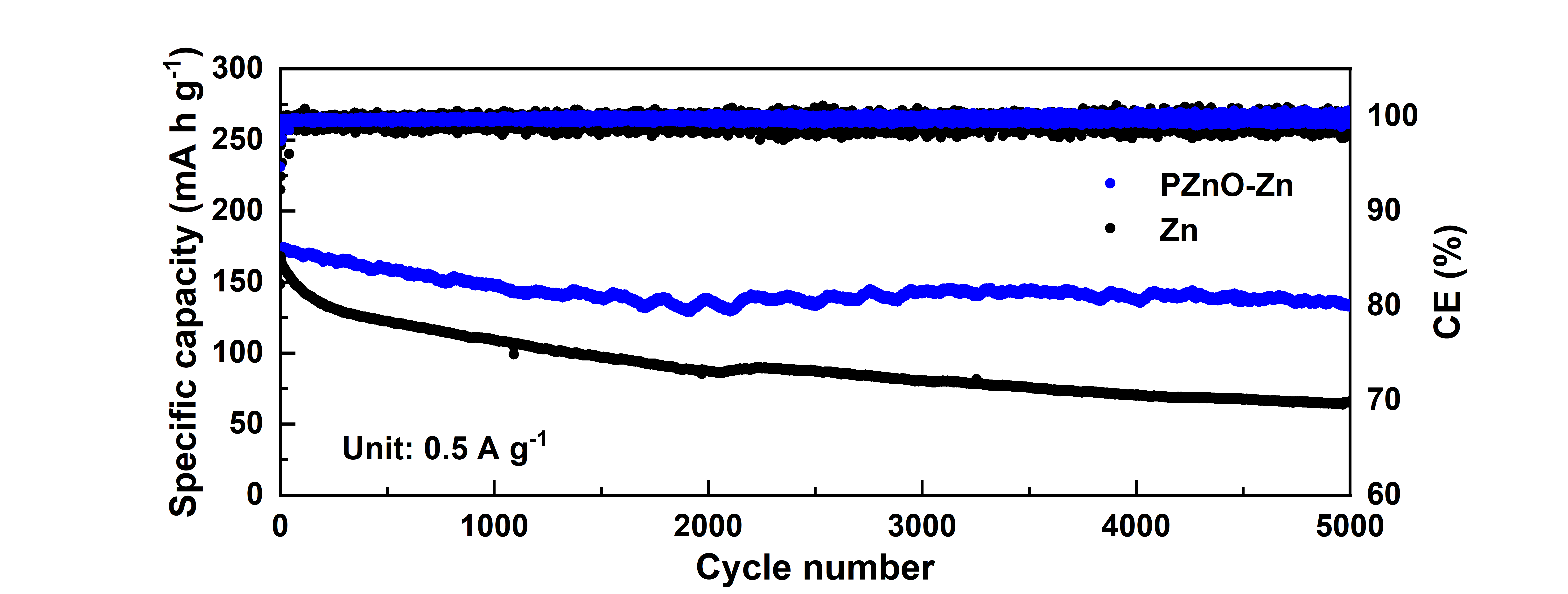


**Fig. S35** Long cycling tests of PZnO-Zn||I_2_ and Zn||I_2_ full cells at 0.5 A g^–1^

**Fig. S36** Long cycling test of PVA-Zn||I_2_ full cells at 2 A g^–1^


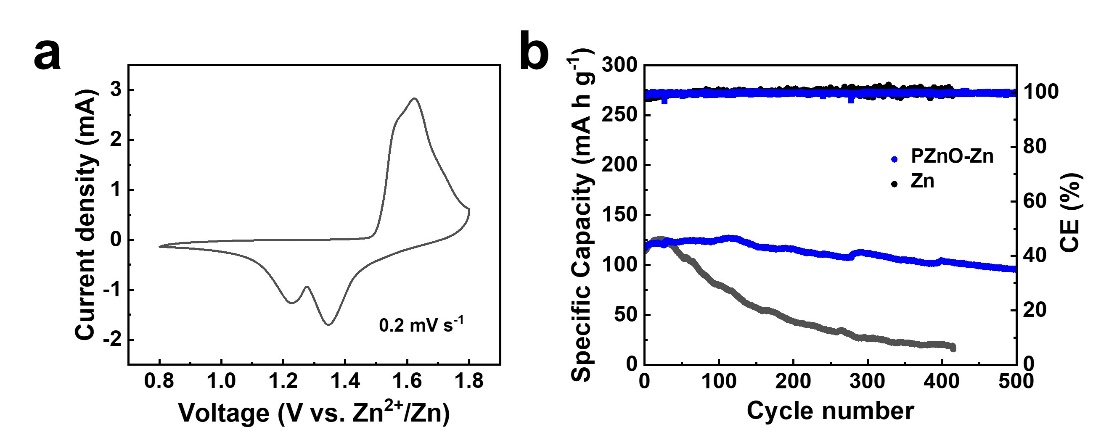


**Fig. S37** (**a**) CV curve of MnO_2_||PZnO-Zn full cell at 0.2 mV s^–1^. (**b**) Cycling stability of MnO_2_||PZnO-Zn (blue) and MnO_2_||Zn (black) full cells at 2C

**Table S1** The comparison of cycling stability of PZnO-Zn with other reported Zn anodes

| Sample | Current density  (mA cm^–2^) | Area capacity  (mA h cm^–2^) | Time  (h) | References |
| --- | --- | --- | --- | --- |
| ZnO/PVA | 1  5  5  10 | 1  1  5  10 | 6000  1700  1600  450 | This work |
| PVA | 0.25  1  5  5  10 | 0.25  1  1  5  5 | 5000  2200  450  300  300 | [S1] |
| MXene/PVA | 5  20 | 1  1 | 400  200 | [S2] |
| 3D ZnO | 5 | 1.25 | 500 | [S3] |
| ZnO | 5  5  10 | 1  2  1 | 1675  675  1126 | [S4] |
| ZnO | 1  5  5  5 | 1  1  2.5  5 | 3100  1600  500  420 | [S5] |
| ZnMoO_4_/PVA | 2  5  10 | 2  5  10 | 2000  1700  275 | [S6] |
| Zn_y_O_1−x_F_x_ | 1  2  5 | 1  1  5 | 1000  1000  250 | [S7] |
| Bi/Bi_2_O_3_ | 1  5  10 | 1  2.5  10 | 3120  750  300 | [S8] |
| Dopamine-functionalized polypyrrole | 1  10 | 1  5 | 1200  300 | [S9] |
| (Ca_5_(PO_4_)_3_F | 1  4  8 | 0.5  1  1 | 4000  2000  2000 | [S10] |
| MXene | 5  10 | 1  1 | 650  480 | [S11] |

**Supplementary References**

1. X. Chen, W. Li, S. Hu, N.G. Akhmedov, D. Reed et al., Polyvinyl alcohol coating induced preferred crystallographic orientation in aqueous zinc battery anodes. Nano Energy **98**, 107269 (2022). <https://doi.org/10.1016/j.nanoen.2022.107269>
2. J. Zhou, Y. Mei, F. Wu, Y. Hao, W. Ma et al., Regulated ion/electron-conducting interphase enables stable zinc-metal anodes for aqueous zinc-ions batteries. Angew. Chem. Int. Ed. **62**(29), e202304454 (2023). <https://doi.org/10.1002/anie.202304454>
3. X. Xie, S. Liang, J. Gao, S. Guo, J. Guo et al., Manipulating the ion-transfer kinetics and interface stability for high-performance zinc metal anodes. Energy Environ. Sci. **13**(2), 503–510 (2020). <https://doi.org/10.1039/C9EE03545A>
4. Q. Ren, X. Tang, K. He, C. Zhang, W. Wang et al., Long-cycling zinc metal anodes enabled by an *in situ* constructed ZnO coating layer. Adv. Funct. Mater. **34**(13), 2312220 (2024). <https://doi.org/10.1002/adfm.202312220>
5. C. Ma, K. Yang, S. Zhao, Y. Xie, C. Liu et al., Recyclable and ultrafast fabrication of zinc oxide interface layer enabling highly reversible dendrite-free Zn anode. ACS Energy Lett. **8**(2), 1201–1208 (2023). <https://doi.org/10.1021/acsenergylett.2c02735>
6. A. Chen, C. Zhao, J. Gao, Z. Guo, X. Lu et al., Multifunctional SEI-like structure coating stabilizing Zn anodes at a large current and capacity. Energy Environ. Sci. **16**(1), 275–284 (2023). <https://doi.org/10.1039/D2EE02931F>
7. S. Zhao, Y. Zhang, J. Li, L. Qi, Y. Tang et al., A heteroanionic zinc ion conductor for dendrite-free Zn metal anodes. Adv. Mater. **35**(18), e2300195 (2023). <https://doi.org/10.1002/adma.202300195>
8. X. Tian, Q. Zhao, M. Zhou, X. Huang, Y. Sun et al., Synergy of dendrites-impeded atomic clusters dissociation and side reactions suppressed inert interface protection for ultrastable Zn anode. Adv. Mater. **36**(19), 2400237 (2024). <https://doi.org/10.1002/adma.202400237>
9. X. Sun, X. Lv, M. Zhang, K. Shi, Z. Li et al., Construction of selective ion transport polymer at anode–electrolyte interface for stable aqueous zinc-ion batteries. ACS Nano **18**(11), 8452–8462 (2024). <https://doi.org/10.1021/acsnano.3c13127>
10. Z. Shi, M. Yang, Y. Ren, Y. Wang, J. Guo et al., Highly reversible Zn anodes achieved by enhancing ion-transport kinetics and modulating Zn (002) deposition. ACS Nano **17**(21), 21893–21904 (2023). <https://doi.org/10.1021/acsnano.3c08197>
11. H. Liu, Z. Xu, B. Cao, Z. Xin, H. Lai et al., Marangoni-driven self-assembly MXene as functional membrane enables dendrite-free and flexible zinc–iodine pouch cells. Adv. Energy Mater. **14**(26), 2470109 (2024). <https://doi.org/10.1002/aenm.202470109>
